# Gut microbiota alterations modulate high-fat diet-induced precocious puberty

**DOI:** 10.1128/spectrum.03264-24

**Published:** 2025-08-12

**Authors:** Nan Wu, Xin Jiang, Yihui Wang, Meilu Zhang, Min Yue, Fei Chen, Wei Wu, Yanan Liu, Qinghua Wang, Lei Zhang

**Affiliations:** 1 Microbiome-X, School of Public Health, Cheeloo College of Medicine, Shandong University 12589, Jinan, China; 2 Department of Psychology, University of California, Santa Cruz, USA; 3 Jinan Institute of Child Health Care, Children's Hospital Affiliated to Shandong University (Jinan Children's Hospital) 576219 , Jinan, China; 4 School of Biological Science and Technology, University of Jinan 12413, Jinan, China; 5 State Key Laboratory of Microbial Technology, Shandong University 520252, Qingdao, China; University of Valencia, Paterna, Valencia, Spain

**Keywords:** gut microbiota, precocious puberty, high-fat diet, metabolomics, microbiome

## Abstract

The link between gut microbiota and precocious puberty has garnered increasing attention; however, the underlying mechanisms remain poorly understood, and most existing studies are cross-sectional, making it difficult to capture the longitudinal changes in gut microbiota throughout precocious puberty. Here, female rats were fed high-fat diets supplemented with antibiotics to establish a model of precocious puberty with gut microbiota perturbation. Gut microbiota dynamics were characterized based on stool samples collected at postnatal day (PND) 21, 25, 29, and 33. Serum metabolomic profiles on PND 33 were analyzed to investigate the potential mechanisms by which gut microbiota may influence precocious puberty. We found that alterations in gut microbiota alleviated high-fat diet-induced precocious puberty. Temporal changes in gut microbiota that potentially regulate precocious puberty were identified, with specific contributions from *Lactobacillus intestinalis*, *Romboutsia*, *Lactobacillus*, and others. Additionally, the modified gut microbiota ameliorated the dysregulation of steroid hormones, bile acids, and lipid molecules at PND 33. Notably, lysoPE 16:0, 23-nordeoxycholic acid, and psoralidin were not only influenced by the gut microbiota but also closely associated with indicators of pubertal development. This study highlights key gut microbiota and metabolites that may inform future mechanistic studies and intervention strategies for managing precocious puberty.

**IMPORTANCE:**

Previous studies have highlighted the link between gut microbiota and precocious puberty. Our research further explored this connection, demonstrating that microbial shifts modulated the onset and progression of precocious puberty, characterized by increased hypothalamic sirtuin-1 gene expression and decreased kisspeptin-1 and gonadotropin-releasing hormone gene expressions. At the same time, we explored the longitudinal trajectories of the gut microbiota and identified the key microbes involved in regulating precocious puberty, including *Romboutsia*, *Lactobacillus*, and others. In addition, an integrated analysis of the microbiome and metabolome revealed specific bacteria and metabolites that contribute to the onset of puberty. These findings offer valuable insights into the mechanisms driving precocious puberty and may inform future research and potential therapeutic interventions.

## INTRODUCTION

Precocious puberty is a common pediatric endocrine disorder characterized by premature secondary sexual characteristics and accelerated physical growth, indicating an abnormal progression of pubertal processes (1). Epidemiological studies show that precocious puberty affects girls at significantly higher rates—approximately 15–20 times more frequently than boys (2). This condition can lead to reduced adult height, an increased risk of various diseases in adulthood, and substantial economic burdens on patients and society due to the high costs of therapy (1, 2). Consequently, there is an urgent need to further investigate the pathogenic factors contributing to precocious puberty and to elucidate the underlying mechanisms, particularly in females.

The current understanding of pubertal pathogenesis highlights the kisspeptin-1 (KISS1) gene as a crucial regulator of reproductive function, which activates the hypothalamic-pituitary-gonadal (HPG) axis. Specifically, kisspeptin, the protein product of KISS1, binds to its receptor GPR54 (KISS1R), leading to the release of gonadotropin-releasing hormone (GnRH) from hypothalamic neurons. This cascade promotes the secretion of luteinizing hormone (LH) and follicle-stimulating hormone from the pituitary gland, driving the onset and progression of precocious puberty (3). Additionally, sirtuin-1 (SIRT1), an NAD(+)-dependent histone deacetylase, interacts with the Polycomb silencing complex at the KISS1 promoter, inhibiting KISS1 expression and subsequently suppressing the HPG axis activation and pubertal onset (4). Despite these insights, external factors influencing hypothalamic gene expression remain largely unknown.

A growing body of recent studies suggests that gut microbiota and its metabolites play a crucial role in the onset and development of precocious puberty. Cross-sectional research has revealed that girls with precocious puberty exhibit decreased abundances of *Bacteroides* and *Faecalibacterium*, along with increased abundances of *Romboutsia*, *Erysipelatoclostridium*, *Roseburia*, and *Tuzzerella* (5, 6). Moreover, fecal microbiota transplantation (FMT) from high-fat diet (HFD)-induced precocious puberty rats led to an earlier onset of puberty and elevated levels of deoxycholic acid (DCA) in recipient rats compared to those receiving FMT from healthy controls (4). Additionally, gut microbiota-derived short-chain fatty acids have been found to alleviate precocious puberty by lowering hypothalamic GnRH and KISS1 expression (7), indicating that gut microbiota may interact with the host’s brain via metabolic pathways. This emerging evidence positions gut microbiota as a potential target for understanding pubertal mechanisms and developing intervention strategies to decelerate pubertal progression. However, the metabolic changes induced by gut microbiota and its interactions in precocious puberty remain poorly understood. Furthermore, most studies have been cross-sectional, leaving a gap in our understanding of the longitudinal changes in gut microbiota during the progression of precocious puberty.

**Fig 1:**
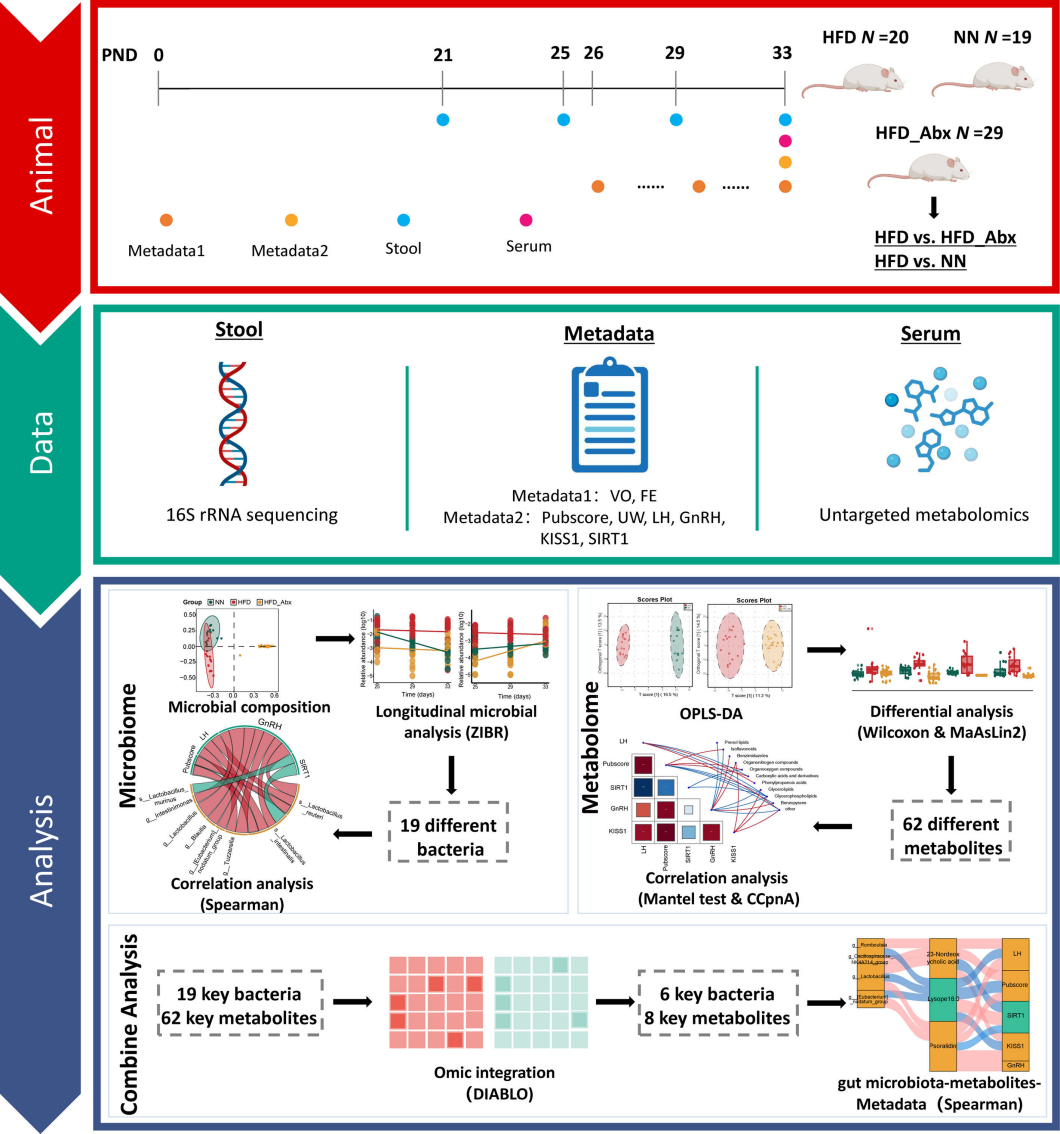
Overview of the workflow. We included HFD, NN, and HFD_Abx groups, with primary comparisons between HFD/NN and HFD/HFD_Abx groups. Fecal samples were collected at PND 21, 25, 29, and 33 and subjected to 16S rRNA sequencing, while blood samples collected at PND 33 underwent untargeted metabolomics. For pubertal development indicators, parameters such as Pubscore, UW, LH, GnRH, KISS1, and SIRT1 were measured at PND 33, and others (VO, FE) were monitored continuously from PND 26 to 33. Subsequently, through bioinformatics and multi-omics analyses, we identified six bacterial taxa and eight metabolites as representative features and further elucidated the interconnected relationships among gut bacteria, metabolites, and indicators of pubertal development.

Our study addresses these gaps by analyzing the dynamic changes in gut microbiota during the onset and progression of precocious puberty using 16S ribosomal RNA (16S rRNA) sequencing at multiple time points. Additionally, we identified metabolites influenced by gut microbiota using cross-sectional untargeted metabolomics. Through integrated analysis of multi-omics data, we explored the potential impact of microbiota-metabolite interactions on biomarkers related to precocious puberty (Fig. 1).

## RESULTS

### Altered gut microbiota alleviates symptoms of precocious puberty in female rats

**Fig 2:**
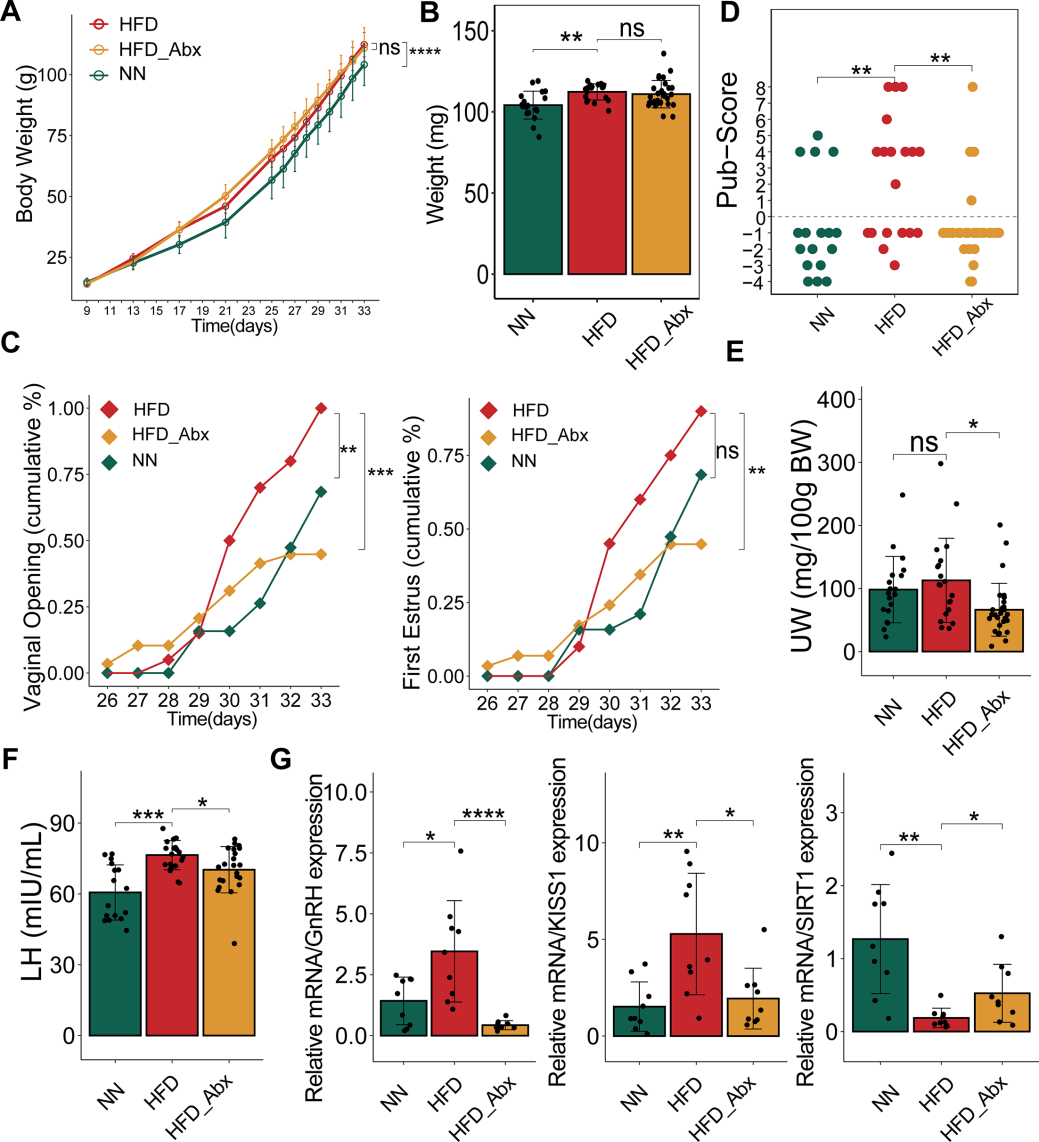
The gut microbiota is critical for the HFD-caused precocious puberty. (**A**) Evolution of body weight. (**B**) Body weight at PND 33. (**C**) Cumulative percentages of VO and FE. (**D**) Histological score of follicular development and ovulation. (**E**) Relative uterine weight. (**F**) Serum LH levels in the same animals at PND 33. (**G**) The mRNA expression of GnRH, KISS1, and SIRT1 in the hypothalamus at PND 33. LME (**A**), Chi-square test or Fisher’s exact test (**C**) and two-tailed Wilcoxon rank-sum test (**B, D–G**) were used for statistical analysis with Benjamini-Hochberg adjustment. *adj.p < 0.05; **adj.p < 0.01; ***adj.p < 0.001; ****adj.p < 0.0001; ns.*adj.p* > 0.05. Total group sizes were HFD  =  20, NN  =  19, HFD_Abx  =  29, while phenotypic and hormonal parameters were assayed in the whole groups, hypothalamic RNA analyses (Fig. 2G) were conducted in a representative subset of randomly assigned samples from each group, with the following distribution: *n* = 9 per group.

To assess the role of the gut microbiota in precocious puberty, we established three experimental groups: HFD, normal diet (NN), and HFD combined with antibiotics treatment (HFD_Abx). Because the NN and HFD_Abx groups incorporated two variables—diet and antibiotics (Abx)—we primarily focused on the comparative results between the HFD and NN groups, as well as between the HFD and HFD_Abx groups. The HFD group exhibited significantly greater weight gain than the NN group (*β* = 0.324, 95% CI: 0.190–0.459, *adj.p* < 0.0001), with no significant difference between the HFD and HFD_Abx groups (*β* = −0.057, 95% CI: −0.133–0.019, *adj.p* = 0.140), consistent with the body weight results at postnatal day (PND) 33 (Fig. 2A and B). However, the cumulative rates of vaginal opening (VO, *adj.p* = 0.0003) and first estrus (FE, *adj.p* = 0.0069) were significantly decreased in the HFD_Abx rats compared to the HFD rats. An increase in cumulative VO rates was observed only in the HFD group relative to the NN group (*adj.p* = 0.0083, Fig. 2C). Additionally, the ovulation rate in the HFD group (57.9%) was higher than that in the NN group (23.5%) and the HFD_Abx group (13.8%). Histological scores indicated that Abx treatment substantially attenuated the adverse effect of HFD on ovarian development (Fig. 2D; Fig. S1B). HFD_Abx rats showed substantially decreased levels of relative uterus weight (UW) although only an upward trend in UW levels was observed in HFD rats (Fig. 2E). Furthermore, Abx supplementation regulated the activity of the HPG axis, as evidenced by decreased serum LH levels, reduced hypothalamic expression of GnRH and KISS1, and elevated hypothalamic SIRT1 mRNA levels (Fig. 2F and G). The statistical results for continuous variables measured at PND 33 are provided in Table S1.

Collectively, these results suggest that HFD induces precocious puberty in female rats, while Abx-mediated alterations in the gut microbiota alleviate symptoms of precocious puberty, accompanied by altered hypothalamic gene expression related to female pubertal development.

### Gut microbial profiles regulating the progression of precocious puberty

**Fig 3:**
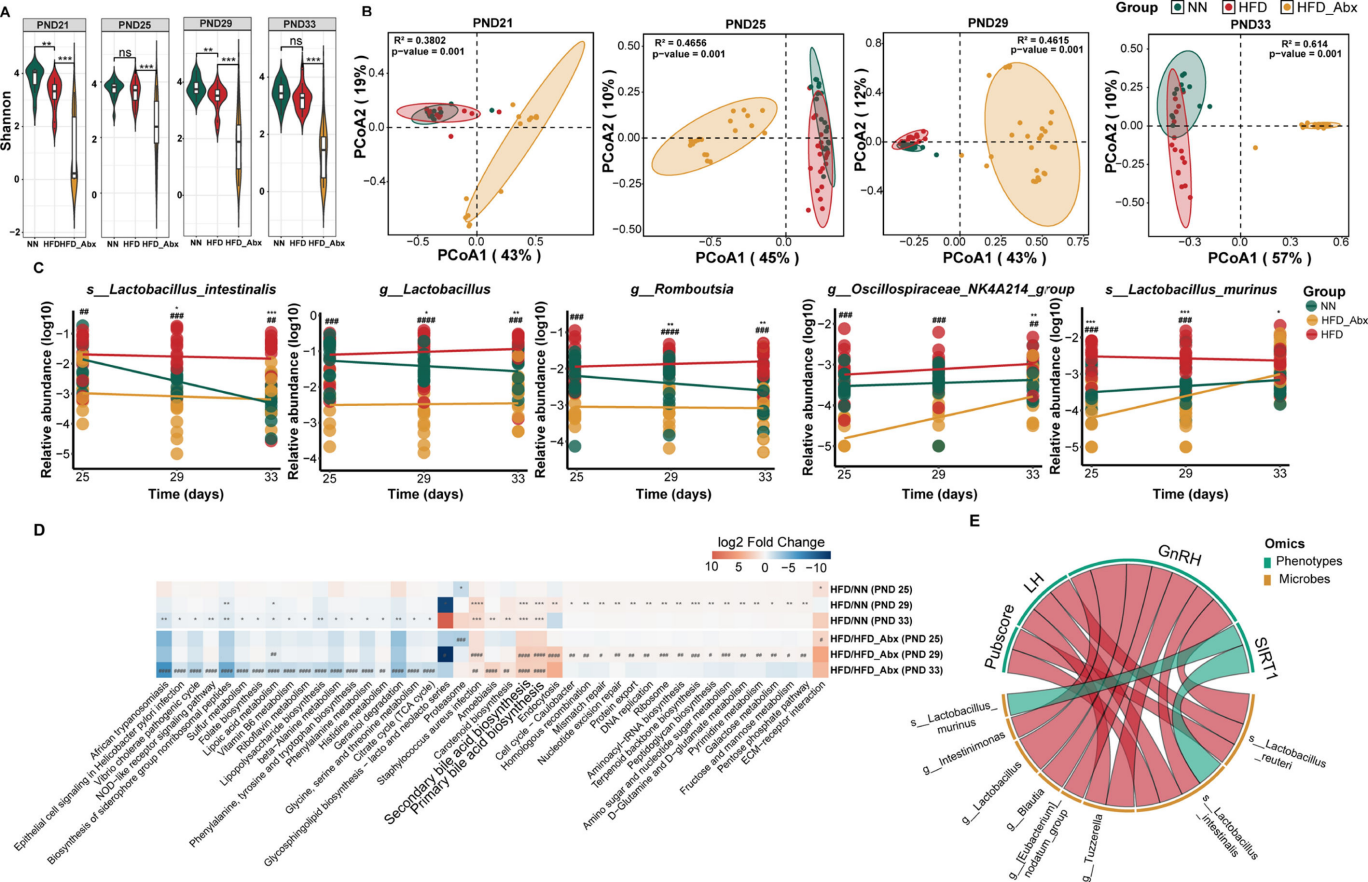
Modifications in microbial profiles caused by Abx treatment. (**A**) Shannon index of the gut microbiome in the HFD, HFD_Abx, and NN groups at four time points. (**B**) Principal coordinate analysis (PcoA), based on Bray-Curtis distance with PERMANOVA, showing the differences in the gut microbiome of stool samples among three groups. (**C**) Changes in relative abundance of *Lactobacillus intestinalis*, *Romboutsia*, *Lactobacillus*, *Oscillospiraceae NK4A214* group, and *Lactobacillus murinus* over time. (**D**) Heatmap showing the abundance differences between the two comparison groups (HFD vs HFD_Abx and HFD vs NN) across the various study timepoints. (**E**) Circus correlation plot showing the correlation between 5 indicators related to precocious puberty and 19 identified bacteria in three groups at PND 33. ZIBR (**C**), two-tailed Wilcoxon rank-sum test (**C and D**) and Spearman rank correlation (**E**) were used for statistical analysis with Benjamini-Hochberg adjustment. The comparison between the HFD and NN groups is represented by *, while # represents the comparison between the HFD and HFD_Abx groups. *(#) *adj.p* < 0.05; **(##) *adj.p* < 0.01; ***(###) *adj.p* < 0.001; ****(####) *adj.p* < 0.0001. Total group sizes were HFD  =  16, NN  =  12, HFD_Abx  =  22.

To identify locally prevalent gut microbiota that potentially modulate the development of precocious puberty, we analyzed stool samples collected at PND 21, PND 25, PND 29, and PND 33 from three groups using 16S rRNA amplicon sequencing. The microbial composition at the genus level revealed that *Lactobacillus* and *Romboutsia* were the dominant genera in HFD rats over time, both of which were less abundant after Abx treatment (Fig. S2A). Alpha diversity indices (Chao1 and Shannon) showed a significant reduction in overall diversity and species richness in the HFD_Abx group compared to the HFD group, while differences between the HFD and NN groups remained at least partially significant at all time points (Fig. 3A; Fig. S2B). For *β* diversity, distinct clustering was formed among the groups over time, with biological replicates well clustered within each group (Fig. 3B). These results suggest that Abx treatment effectively diminishes gut microbiota diversity and alters community structure.

Given that PND 21 was the weaning time point and that numerous factors affected the gut microbiota, subsequent analyses focused on data from the other three time points. Longitudinal microbial analysis identified 19 shared differentially abundant genera and species between the two comparison groups (HFD/NN, HFD/HFD_Abx), which were recognized as risk factors for the development of precocious puberty and showed reduced abundances following Abx treatment (Table S2). Among the aforementioned bacteria, both precocious puberty-associated taxa (e.g., *Tuzzerella*, *Erysipelatoclostridium* [8], *Roseburia* [5], *Bilophila*, *Blautia* [9], *Lachnoclostridium*, *Intestinimonas*, *Lactobacillus* [4], *Enterococcus* [10]) and butyrate-producing bacteria (*Butyrivibrio* [11], *Romboutsia* [12], *Erysipelatoclostridium* [13], *Blautia* [14], *Intestinimonas* [15], and *Roseburia* [16]) were observed. Notably, three bacteria—*Lactobacillus intestinalis*, *Romboutsia*, and *Lactobacillus*—consistently showed higher relative abundances in HFD rats, whereas their abundances gradually decreased over time in NN rats. In the HFD_Abx group, their abundances remained relatively stable, likely due to the effects of Abx. Additionally, the *Oscillospiraceae NK4A214* group and *Lactobacillus murinus* maintained high abundances in HFD rats throughout the study, while their abundances showed an increasing trend in NN and HFD_Abx rats (Fig. 3C; Table S3). Recent studies have reported that butyrate-producing bacteria, such as *Romboutsia* and *Oscillospiraceae NK4A214* group, may promote the progression of precocious puberty by enhancing intestinal inflammation and releasing neurotransmission-related metabolites. Meanwhile, *Lactobacillus* and its species can produce bile salt hydrolase (BSH) to regulate bile acid levels, which are closely related to precocious puberty. Furthermore, among the 19 bacteria, *Lactobacillus* (*Lactobacillus reuteri*, *Lactobacillus intestinalis*), *[Eubacterium] nodatum group*, *Tuzzerella*, *Blautia* (4, 9), and *Intestinimonas* (4) were positively correlated with GnRH gene expression. *Lactobacillus* and its species also showed correlations with several indicators (Pubscore, LH, GnRH, and SIRT1) (Fig. 3E).

In our microbial functional prediction analysis at three time points, we identified 23 pathways with higher abundances in the HFD group, which were decreased by altered gut microbiota, while the opposite was found for 20 pathways. Most of these pathways were related to inflammatory reactions, oxidative stress (OS), and neuroendocrine functions, suggesting their potential effects on the development of precocious puberty.

These pathways included biosynthesis of phenylalanine, tyrosine and tryptophan, phenylalanine metabolism, vitamin B6 metabolism (17), histidine metabolism, D-Glutamine and D-glutamate metabolism, nucleotide-binding and oligomerization domain (NOD)-like receptor signaling pathway, lipoic acid metabolism (18), mismatch repair (19), pentose phosphate pathway (20), biosynthesis of siderophore group nonribosomal peptides (21), geraniol degradation (22), and others (Table S4). We also observed that the abundance differences in primary and secondary bile acid biosynthesis between the two comparison groups (HFD/NN, HFD/HFD_Abx) showed an increasing trend (Fig. 3D). Of note, at PND 29, the biosynthesis of siderophore group nonribosomal peptides, primary bile acid biosynthesis, and secondary bile acid biosynthesis were the three nodes with the highest degree of connectivity, which correlated with the abovementioned 19 bacteria. At PND 33, these pathways together with lipoic acid metabolism and geraniol degradation continued to demonstrate significant connectivity with the same bacteria (Fig. S2C). Taken together, these results suggest that the identified gut microbes have the potential to influence the development and progression of precocious puberty by modulating inflammatory reactions, OS, and bile acid levels.

### Gut microbiota modification causes changes in serum metabolomics

**Fig 4:**
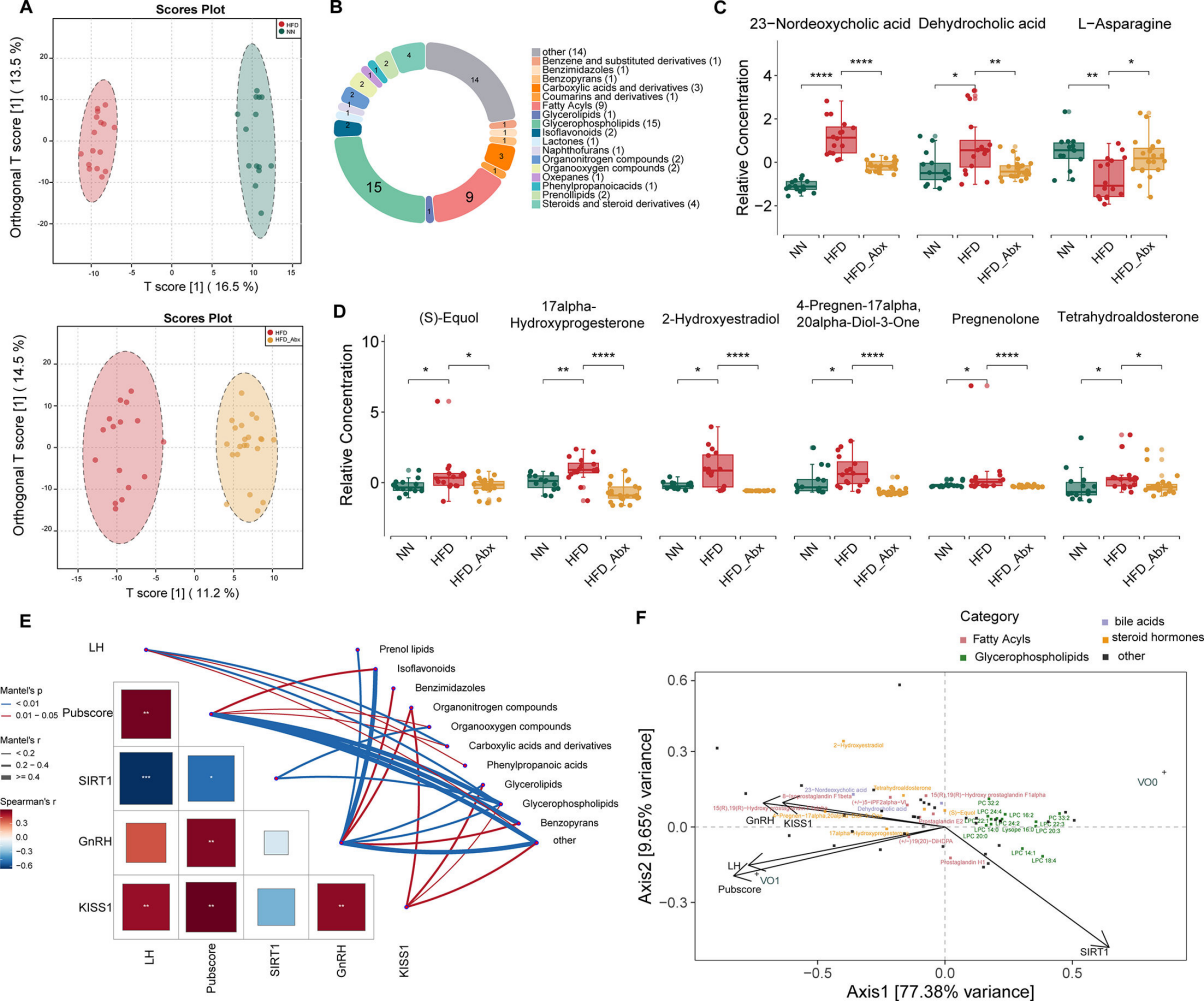
Shifts in gut microbiota affect serum metabolomics in female rats with precocious puberty. (**A**) OPLS-DA of serum metabolites, with the ellipse representing the 95% CI. (**B**) A pie chart illustrating the categories of 62 metabolites based on identification results from the HMDB database. Comparison of bile acid-related metabolites, L-asparagine (**C**), and steroid hormones (**D**) between the two comparison groups (HFD vs HFD_Abx and HFD vs NN). (**E**) The corrplot for the identified five indicators of pubertal maturation and their relations to the identified metabolic categories. (**F**) CCpnA between five indicators of pubertal maturation and differential metabolites, including continuous variables (arrows), categorical variables (+), and metabolites (squares). The two-tailed Wilcoxon rank-sum test (**C and D**) was used for statistical analysis with Benjamini-Hochberg adjustment. *adj.p < 0.05; **adj.p < 0.01; ***adj.p < 0.001; ****adj.p < 0.0001. Total group sizes were HFD  =  13, NN  =  12, HFD_Abx  =  21.

Based on the observed alterations in gut microbial functions, we employed untargeted metabolomics at PND 33 to examine the impact of gut microbiota changes on serum metabolites. The Orthogonal Projection to Latent Structures Discriminant Analysis (OPLS-DA) score plots clearly separated the HFD and NN groups (*R*^2^*X* = 0.295, *R*^2^*Y* = 0.996, *Q*^2^ = 0.933), as well as the HFD and HFD_Abx groups (*R*^2^*X* = 0.306, *R*^2^*Y* = 0.986, *Q*^2^ = 0.883), indicating the global impact of gut microbiota changes on the serum metabolomics (Fig. 4A). Among 1,014 identified metabolites, we focused on shared differentially abundant metabolites in the HFD/NN and HFD/HFD_Abx comparisons, which were significantly altered in the HFD group and reversed by Abx treatment (Fig. S3A). The results identified 62 shared differential metabolites using Wilcoxon and MaAsLin2 analyses, with the most abundant categories related to lipid molecules, such as fatty acyls and glycerophospholipids (GPs) (Fig. 4B; Table S5 and S6). Among these metabolites, altered gut microbiota effectively decreased the levels of OS and inflammation markers (23), as demonstrated by the reduced levels of prostaglandins (PGs) and their derivatives belonging to fatty acyls, and the elevated levels of GPs in HFD rats (Fig. S3B and C). Furthermore, levels of steroid hormones and bile acid-related metabolites—including (S)-equol, 17alpha-hydroxyprogesterone, 4-pregnen-17alpha,20alpha-diol-3-one, pregnenolone, tetrahydroaldosterone, 2-hydroxyestradiol, dehydrocholic acid, and 23-nordeoxycholic acid (norDCA)—significantly decreased with gut microbiota alterations, whereas L-asparagine levels increased (Fig. 4C and D). Kyoto Encyclopedia of Genes and Genomes (KEGG) pathway analysis revealed that the differential metabolites were mainly involved in steroid hormone biosynthesis, fatty acid metabolism, and OS-related pathways, such as glutathione metabolism, arginine and proline metabolism, and arachidonic acid metabolism (Fig. S3D). We further assessed the relationships between the identified metabolites and indicators of pubertal development. GnRH and Pubscore emerged as the two major indicators significantly associated with the metabolites, especially isoflavonoids and GPs, respectively (Fig. 4E). Canonical correspondence analysis (CCpnA) also revealed that the puberty indicators (VO, LH, Pubscore, GnRH, and KISS1) were inversely correlated with GPs and positively correlated with fatty acyls, steroid hormones, and bile acid-related metabolites, whereas SIRT1 displayed an opposite relationship (Fig. 4F).

### Interactions between altered gut microbiota and serum metabolites affect the development of precocious puberty

**Fig 5:**
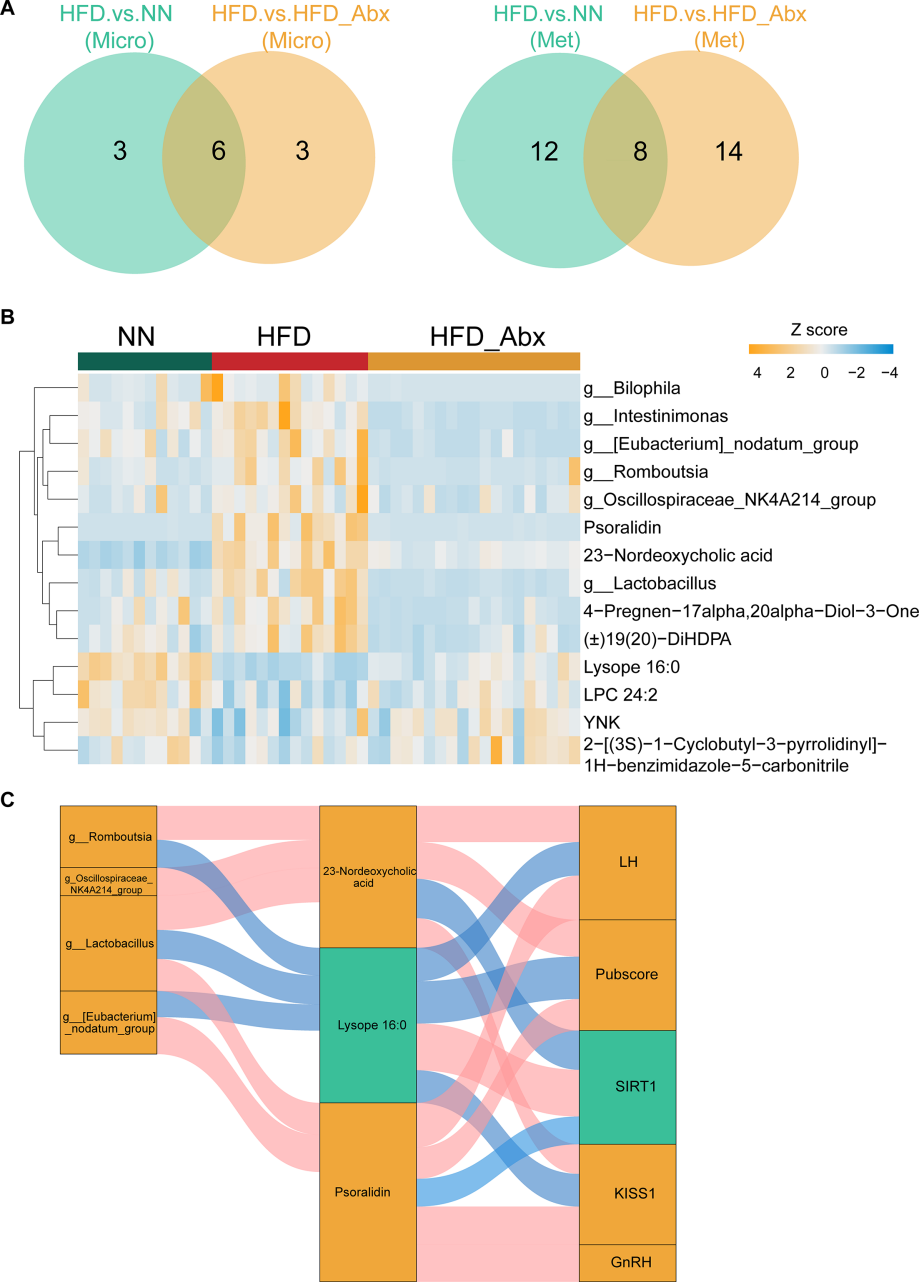
Multi-omics integration of gut microbiota and metabolomics profiling. (**A**) The shared candidate microbial and metabolic biomarkers in NN and HFD_Abx compared to the HFD groups using Venn analysis. (**B**) Heatmap displaying the most contributing microbial and metabolic molecules for the first two components. (**C**) Sankey plot illustrating the Spearman correlation analysis between gut microbiota, metabolites, and variables associated with precocious puberty.

To further identify the key molecules involved in gut microbiota-mediated regulation of precocious puberty, a multi-omics integrated analysis using the Data Integration Analysis for Biomarker Discovery using Latent Components (DIABLO) method was performed in two comparison groups (HFD/NN, HFD/HFD_Abx). The results showed clear discrimination between two groups based on the key characteristics of gut microbiota and metabolites (Fig. S4A and B). The distinctive signatures for each omic were shown in Fig. S4C and D. Eight metabolites and six bacteria had a strong contribution to the discrimination between the groups (Fig. 5A). Specifically, Abx treatment led to lower levels of multiple gut microbes and metabolites, including *Bilophila*, *Intestinimonas*, *[Eubacterium] nodatum group*, *Romboutsia*, *Lactobacillus*, *Oscillospiraceae NK4A214* group*,* psoralidin (PSO), norDCA, 4-pregnen-17alpha,20alpha-diol-3-one, (±)19(20)-DiHDPA. Conversely, lysoPE 16:0, LPC 24:2, YNK, and 2-[(3S)-1-cyclobutyl-3-pyrrolidinyl]-1H-benzimidazole-5-carbonitrile exhibited an opposite trend (Fig. 5B). Finally, the potential relationships between gut microbiota, metabolites, and indicators of pubertal development were investigated. The metabolites were not only associated with gut microbiota, but were also closely related to indicators of precocious puberty (Fig. 5C; Table S7). As a risk factor for precocious puberty, norDCA was linked to an increased abundance of *Romboutsi*a, *Oscillospiraceae NK4A214* group, and *Lactobacillus*, as well as elevated levels of LH, Pubscore, and KISS1. SIRT1 levels were found to be lower in this context. Psoralidin demonstrated a positive relationship with ovarian development and elevated LH and hypothalamic gene expression, including GnRH and KISS1. This correlation was consistent with increased levels of the *[Eubacterium] nodatum group* and *Lactobacillus*. In contrast, lysoPE 16:0, a beneficial metabolite, tended to decrease alongside the abundance of *[Eubacterium] nodatum group*, *Lactobacillus*, *Romboutsia*, and was inversely correlated with LH levels, KISS1 expression, and ovarian maturity.

## DISCUSSION

Recent research has highlighted the gut microbiota as a key factor in the development of precocious puberty (4). However, most of these studies have been cross-sectional, leaving the mechanistic relationship between the gut microbiota and precocious puberty unclear. To address this gap, we treated female rats with precocious puberty using Abx to alter gut microbiota, followed by longitudinal microbiome profiling and integration of multidimensional data sets from the fecal microbiome and serum metabolome analyses, to gain a more profound understanding of the gut microbiota and explore potential mechanisms. Our findings revealed that alterations in the gut microbiota delayed the onset of puberty by modulating metabolic disruptions that might accelerate OS and influence the levels of bile acids and steroid hormones, thereby impacting the progression of precocious puberty. Key microbial and metabolomic contributors to the gut microbiota-mediated regulation of precocious puberty were also identified.

Our experimental design focused on PND 33 as the primary endpoint for mechanistic investigations based on three critical considerations. First, VO—a reliable marker for pubertal onset—was completed in all HFD rats by PND 33, with significantly different cumulative VO rates compared to the NN (68.42%) and HFD_Abx (44.83%) groups (*adj.p* < 0.01), effectively capturing intergroup differences (24). Second, LH levels, a direct indicator of HPG axis activation (25), peak at VO and exhibit minimal post-pubertal fluctuations (26). Delayed euthanasia would blur intergroup differences in LH and hypothalamic gene expression, as rats in the NN and HFD_Abx groups enter puberty sequentially. Third, our modeling approach and euthanasia timing were aligned with the seminal work of Vazquez et al. (27) by conducting terminal analyses at PND 33 following confirmed VO completion across all HFD-treated rats. Additionally, to establish a mechanistic link between the gut microbiota, metabolites, and pubertal regulation, we collected tissue, serum, and fecal samples at PND 33 to ensure congruence between microbiome and serum metabolome data sets and pubertal markers.

Through longitudinal 16S rRNA sequencing, we identified a gut microbiome-derived signature comprising 19 bacteria that might regulate the progression of precocious puberty. Over time, female rats with precocious puberty exhibited higher abundances of *Romboutsia*, *Lactobacillus,* and *Lactobacillus intestinalis*, while the abundance gradually decreased in normal controls, suggesting their potential harmful effects. Notably, no significant intergroup differences were observed at PND 25 (equivalent to 6 years of age in humans), a phenomenon presumably attributed to the prevalence of these dominant bacteria in young children (28, 29). Additionally, the *Oscillospiraceae NK4A214* group and *Lactobacillus murinus* had consistently higher abundances compared to NN controls although this intergroup difference gradually diminished over time. Given that these taxa typically increase during adolescent and adult stages (30, 31), we postulate that their abundances would normally increase with age, but HFD maintains them at elevated levels prematurely, which may facilitate the onset of precocious puberty. Further investigation is essential to understand the underlying mechanisms involved in this process.

Of particular interest is *Romboutsia*, a butyrate-producing bacterium whose metabolic byproducts are mechanistically linked to heightened inflammation, OS, and neuromodulation. The elevated abundance of *Romboutsia* observed in the HFD group of our study suggests it may contribute to a milieu promoting the progression of precocious puberty. Specifically, butyrate’s documented ability to suppress superoxide dismutase 2 induces OS that may intensify during puberty (32). Our analyses further substantiated this connection, showing that *Romboutsia* was closely linked to inflammatory and pro-oxidative pathways, such as geraniol degradation (22), biosynthesis of siderophore group nonribosomal peptides (21), and vitamin B6 metabolism (17), the last of which was crucial for neurotransmitter synthesis and synaptic transmission (33, 34). Moreover, butyrate has been shown to upregulate inducible nitric oxide synthase (iNOS), thereby enhancing nitric oxide (NO) production. This gaseous neurotransmitter stimulates GnRH secretion and activates the HPG axis (35, 36)—a mechanism supported by clinical observations of elevated NO synthesis activity in girls with central precocious puberty compared to age-matched controls (37). Importantly, our analysis revealed that *Romboutsia* may exacerbate this process through its reduction of lysoPE 16:0, a glycerophospholipid metabolite known to negatively regulate NO levels (38).

*Lactobacillus* is the predominant resident flora in the intestines of newborns, with its abundance declining with age. This partially explains the higher levels observed at PND 25 in HFD and NN groups, while the NN group exhibited a decline over time (28, 29). This microbial population exhibits female-predominant sexual dimorphism and may modulate the HPG axis through regulation of OS homeostasis and bile acid metabolism. Specifically, *Lactobacillus* encodes BSH, an enzyme that directly influences bile acid profiles (12, 39). Our data support this link, demonstrating temporal increases in the enrichment of *Lactobacillus*-associated primary and secondary bile acid biosynthesis pathways in HFD rats, and a positive correlation between *Lactobacillus* and norDCA. These findings suggest that *Lactobacillus* influences the onset of puberty through bile acid-dependent pathways. Notably, different bile acids may exert divergent effects on pubertal development. For instance, the secondary bile acid glycodeoxycholic acid (GDCA) attenuates the progression of precocious puberty by suppressing the activity of the HPG axis (40), whereas the bile acid receptor Takeda G protein-coupled receptor 5 (TGR5)—primarily activated by lithocholic acid (LCA) and DCA—may stimulate GnRH release through kisspeptin receptors, accelerating the onset of puberty in female rats (41). Additionally, *Lactobacillus* exacerbates OS through pro-oxidant PSO production and increased lactic acid production from lactose fermentation, which collectively disrupt pubertal development (42).

Interestingly, microbes negatively correlated with obesity—such as *Bilophila* (43, 44), *Lachnoclostridium* (45), *Lactobacillus*, *Marvinbryantia* (46), *Lactobacillus murinus* (47), *Lactobacillus reuteri* (48), and *Roseburia* (49)—were enriched in female rats with precocious puberty, suggesting that the microbial communities associated with obesity differ significantly from those linked to pubertal development. Similarly, weight comparisons at PND 33 and across longitudinal timepoints showed that the changes in gut microbiota mitigating precocious puberty were not accompanied by weight changes. The results indicate that weight gain or even obesity may not be a risk factor for developing precocious puberty. Increasing evidence suggests that HFD, rather than obesity, may significantly influence pubertal development. Several cross-sectional and longitudinal epidemiological studies have linked HFD to precocious puberty in children, showing that increased dietary fat intake, particularly monounsaturated fatty acids, is associated with an earlier onset of puberty (50–52). Additionally, animal and *in vitro* studies have reported that excessive intake of saturated fatty acids can trigger hypothalamic inflammation and microglial activation, as well as phoenixin upregulation, thereby promoting an earlier onset of puberty (53–57). In our study, bacteria capable of lipolysis and fatty acid absorption were significantly enriched in female rats with precocious puberty (32, 58, 59), accompanied by changes in bacteria potentially impacting inflammation and OS—such as *Lactobacillus murinus* (60), *Marvinbryantia* (61), *Erysipelatoclostridium* (62), and *Lachnoclostridium* (63)—and this observation supports the aforementioned views.

Metabolomic analyses of serum samples identified that altered gut microbiota decreased the levels of lipid molecules, such as 8-iso-15-keto prostaglandin F2alpha (8-iso-PGF2α), prostaglandin E2, (±)19 (20)-DiHDPA, prostaglandin H1, 8-isoprostaglandin F1beta, (+/−)5-iPF2alpha-VI, and 15(R),19(R)-hydroxy prostaglandin F1alpha (64, 65). These changes may disrupt lipid metabolism and contribute to increased OS. Notably, 8-iso-PGF2α is identified as a potential inducer of OS and is closely associated with the onset of puberty (32). Prostaglandin E2, released by hypothalamic microglia as an inflammatory mediator, has been shown to induce GnRH secretion in a dose-dependent manner (66). We also found alterations in steroid hormones, such as (S)-equol, 17alpha-hydroxyprogesterone, 4-pregnen-17alpha,20alpha-diol-3-one, pregnenolone, tetrahydroaldosterone, and 2-hydroxyestradiol. In contrast, levels of L-asparagine, as a neurotransmitter, were increased after gut microbiota alteration. Lin et al. (67) found that long-term consumption of aspartame, which consisted of aspartate and phenylalanine, delayed the onset of puberty in female rats by the inhibition of the HPG axis. The results indicate that a distinct distribution of several metabolites related to steroid hormones, neurotransmitters, and OS in circulation plays a key role in the process of gut microbiota-regulated precocious puberty.

This study identified a potential beneficial metabolite, lysoPE 16:0, which was positively correlated with SIRT1, KISS1, LH, and Pubscore, whereas norDCA and PSO showed opposite correlations. All three metabolites are influenced by gut microbiota. As a host-derived lysophospholipid generated via phospholipase A2 (PLA2)-mediated deacylation of phosphatidylethanolamine (68), lysoPE 16:0 was demonstrated to be collectively associated with *Eubacterium nodatum group*, *Romboutsia* (likely through production of acetate and butyrate), and *Lactobacillus* (via modulating bile acid metabolism), all of which have the capacity to influence PLA2 activity (69–71). Mechanistically, lysoPE 16:0 reduces NO production by inhibiting iNOS (38), thereby blocking the NO-mediated cascade that stimulates GnRH secretion and LH surge (72). Notably, this study first reported positive correlations between lysoPE 16:0 and both SIRT1 and KISS1, suggesting a potential role in modulating KISS1 expression through SIRT1-dependent mechanisms. Additionally, norDCA, a gut microbiota-derived metabolite of DCA associated with *Lactobacillus* and *Romboutsia* with BSH activity (73, 74), acts as a farnesoid X receptor (FXR) agonist to promote precocious puberty through dual regulation: centrally, it modulates hepatic receptor homologue-1-mediated transcriptional activation of KISS1 in hypothalamic arcuate neurons, thereby elevating serum LH levels (75); gonadally, it coordinately upregulates steroidogenic enzymes (CYP19a1 and HSD17b1) while downregulating the estrogen-metabolizing enzyme (SULT1E1), resulting in endogenous estrogen accumulation (76, 77). This is consistent with our finding of positive correlations between norDCA and KISS1 gene expression, LH levels, and Pubscore. Our study also revealed that PSO, a bioactive compound metabolized by gut microbiota, was positively correlated with the relative abundances of *Eubacterium nodatum group* and *Lactobacillus* (78). PSO may promote precocious puberty through multiple pathways: first, acting as an estrogen receptor agonist to upregulate hypothalamic KISS1 gene expression and induce microglial production of proinflammatory cytokines for GnRH neuronal activation (75, 79); second, increasing hypothalamic serotonin (5-HT) levels to stimulate RP3V KISS1 and GnRH neurons through 5-HT2 receptor-mediated signaling (80–82); third, disrupting serum corticotropin-releasing factor (CRF) levels to relieve the CRF-mediated inhibition of the HPG axis (80, 83).

This study has certain limitations. First, while we have identified the associations among gut microbiota, metabolites, and precocious puberty, the causal mechanisms require further validation through *in vivo* (e.g., FMT, gnotobiotic models, bacterial mono-colonization, and metabolic interventions) and *in vitro* (bacterial monocultures, bacterial cultures with host cells, and hypothalamic-pituitary cell line assays) approaches, complemented by intestinal fluid metabolomics for more accurate delineation of microbiota-derived metabolites. Second, the absence of an NN combined with Abx treatment (NN_Abx) group limits differentiation between antibiotic-specific effects and HFD-antibiotic interactions. Existing evidence shows that HFD both modifies gut microbiota and its metabolite profile and promotes the transfer of antibiotic resistance genes, thereby enhancing host tolerance to Abx and reducing antibiotic efficacy (84, 85). Moreover, Abx alone may regulate the pubertal development in normal rodents by modulating SCFAs, bile acid profiles, and metabolic hormones (7, 40, 86–88). Comparing NN and NN_Abx groups would clarify whether the antibiotic-mediated alleviation effects are attributed to HFD-dependent (in the absence of group differences) or HFD-independent (in the presence of significant differences) effects. Finally, future research should incorporate longitudinal analyses to elucidate the specific role of gut microbiota in maternal HFD-induced precocious puberty in female offspring and to explore effective dietary strategies through switching from HFD to NN during pregnancy and lactation.

In conclusion, we identified distinct temporal variations in the gut microbiota that has the potential to influence the process of precocious puberty, including *Romboutsia*, *Lactobacillus, Lactobacillus intestinalis,* and others. Our multi-omics analyses identified potential mechanisms by which the altered gut microbiota may influence the levels of bile acids and OS, thereby delaying the onset of puberty. The longitudinal design of our study enables identification of microbial temporal dynamics associated with the development of precocious puberty not detectable by prior cross-sectional studies. This study also provides valuable insights into the complex interplay between gut microbiota and metabolites in the development of precocious puberty and warrants further investigation.

## MATERIALS AND METHODS

### Animals

Eighteen female Wistar rats (215–250 g) and nine male Wistar rats (330–360 g), all aged 9 weeks, were purchased from Beijing Vital River Laboratory Animal Technology Co., Ltd. (Beijing, China) for breeding to obtain female offspring. All rats were housed at the Animal Center of Shandong Normal University under controlled conditions: a constant room temperature of 22°C, humidity between 50% and 60%, and a consistent light cycle (14 h of light per day, starting at 7 a.m.). The rats had free access to water and food. The animal experiments were approved by the Ethics Committee on Public Health of Shandong University (ethical approval number LL202303027).

Female Wistar pups aged on PND 10 were randomly divided into three groups: HFD (*n* = 20), HFD_Abx (*n* = 29), and NN (*n* = 19). This design aimed to establish models of HFD-induced precocious puberty, gut microbiota-altered precocious puberty, and normal controls. The rats were euthanized on PND 33, and serum, hypothalamus, ovaries, uterus, and fecal samples were collected for analysis.

In the HFD and HFD_Abx models, the pups were housed in small cages (4 pups per dam) from PND 10 to PND 20. From PND 21 to PND 33, the female rats were fed HFD (D12451, Research Diets, USA) (27). Between PND 11 and PND 31, each female rat in the HFD_Abx model was administered a daily antibiotic cocktail consisting of 10 mg neomycin sulfate, 10 mg vancomycin hydrochloride, 10 mg ampicillin, and 5 mg metronidazole, all provided by Sangon Biotech (China). The HFD model was given phosphate-buffered saline (PBS) in the same volume as the antibiotic mixture used in the HFD_Abx model. In contrast, for the NN model, the pups were housed in standard cages (12 pups per dam) and were breastfed. From PND 21 to PND 33, the rats were fed a normal control diet (D12450B, Research Diets, USA). From PND 11 to PND 31, the rats were gavaged with the same volume of PBS. The flow chart of animal treatments is presented in Fig. S1A.

### Measurements of the body weight and uterine weight

The body weight of the rats was measured and recorded every 4 days from PND 10 to 20. From PND 21 until the end of the experiment, each rat’s weight was recorded daily. A linear mixed-effects model (LME) was used to analyze the differences in body weight growth trends among three groups of rats, where the fixed effects included PND, group, and the interaction between PND and group (PND × group), while the random effect was the rat’s identification number. Uterine samples were collected for weighing and subsequently standardized to mg/100 g of body weight.

### Assessment of pubertal development

The assessment of pubertal development included the following components: (a) determination of the VO time and the onset of the FE and (b) evaluation of ovarian development and ovulatory function.

Based on previous data regarding the normal timing of puberty in female rodents, each female rat was observed for VO daily from PND 26 to PND 33. If VO was observed, a vaginal smear was immediately collected to assess the estrous cycle.

The collected left ovaries, including the oviducts and tips of the uterine horns, were placed in fixative, dehydrated, embedded in paraffin, sectioned, and stained with hematoxylin-eosin. Ovarian development was then observed and evaluated using an inverted microscope (CKX53, Olympus Corporation, Japan) and quantified using a Pub-Score (24).

### Hormone assays

According to the instructions of the ELISA kits (MEIKE, China), the concentrations of LH in serum samples were measured. The optical density was assessed at a wavelength of 450 nm using a BioTek Epoch 2 Microplate Spectrophotometer (BioTek, USA). The concentrations of hormones in each sample were calculated using a linear regression equation derived from the standard curve analysis.

### Real-time quantitative PCR

First, total RNA was extracted from hypothalamic tissue according to the instructions provided in the Tissue RNA Extraction Kit (Vazyme, China). The RNA was then reverse transcribed into cDNA with the HiScript III RT SuperMix for qPCR Kit (Vazyme, China) under the following conditions: 37°C for 15 min, followed by 85°C for 5 s. Finally, real-time fluorescence quantitative PCR was performed using the ChamQ Universal SYBR qPCR Master Mix Kit (Vazyme, China). DNA was amplified under the following reaction conditions: an initial denaturation at 95°C for 30 s, followed by 40 cycles consisting of denaturation at 95°C for 5 s and annealing/extension at 53°C for 20 s. To ensure reliability, three technical replicates were performed for each sample. The relative expression levels of the genes were calculated using the 2^−ΔΔCT^ method, with glyceraldehyde-3-phosphate dehydrogenase (*GAPDH*) serving as the internal control. The forward and reverse primers were listed in Table S8.

### DNA extraction and 16S rRNA gene sequencing

Fecal samples were processed using the Magnetic Soil and Stool DNA Kit (DP336; TianGen; Beijing; China) to extract total genomic DNA. The extracted DNA underwent quantification and quality assessment to ensure its suitability for sequencing. Utilizing the universal primer pair F341 (5′-CCTAYGGGRBGCASCAG-3′) and R806 (5′-GGACTACNNGGGTATCTAAT-3′), the 16S rRNA gene targeting the variable V3 and V4 regions was amplified. After mixing and purifying the amplified PCR products, the 16S rRNA fragments were used to construct sequencing libraries, which were then subjected to high-throughput sequencing on the Illumina NovaSeq 6000 platform, generating 250 bp paired-end sequencing data.

### Bioinformatics analysis

Quantitative Insights Into Microbial Ecology (QIIME2, version 2020.2) is widely recognized as an effective tool for analyzing microbial ecology and processing raw 16S rRNA sequencing data (89). Quality assessment of the raw data and removal of forward and reverse primers were conducted with FastQC (version 0.11.8) and Cutadapt (version 1.9.1). Subsequently, the DADA2 method was employed to denoise the paired-end sequencing data, conduct quality control, identify and eliminate chimeras, and produce high-quality feature tables along with representative sequences. A species classifier was trained utilizing the Silva database, and the trained classifier was then used to classify the amplicon sequence variants (ASVs) at a 99% similarity threshold (90). Samples and ASVs were filtered as follows: samples with fewer than 10,000 ASV sequences were excluded. Additionally, ASVs with a total abundance (the sum across all samples) of fewer than 20 or those present in only one sample were removed. Mitochondrial and chloroplast sequences were eliminated, retaining only sequences that were annotated at the phylum level. Finally, based on the ASV annotation information, ASVs abundances were integrated to generate abundance data at the taxonomic level.

At each time point (PND 21, PND 25, PND 29, and PND 33), alpha diversity was assessed using the Chao1 and Shannon indices, while beta diversity was evaluated based on the Bray-Curtis distance and tested for statistical significance using permutational multivariate analysis of variance (PERMANOVA). These analyses were conducted using the R package 'MicrobiotaProcess' (version 1.10.3) (91) and 'vegan' (version 2.5.7) (92), respectively. The profiles of the top 10 abundant genera were presented using stacked bar plot at the four different time points.

In cross-sectional and longitudinal analyses of microbial abundance, a two-tailed Wilcoxon rank-sum test and two-part zero-inflated beta regression with random effects (ZIBR) models were employed, with the latter utilizing the R package 'ZIBR' (version 1.0.2) (93). Microbial gene functions were predicted using PICRUSt with the R package 'PICRUSt2' (version 2.5.2), which was based on the KEGG database (94). Correlations between microbial factors and pubertal development factors, as well as between microbial factors and microbial functional pathways, were assessed using Spearman’s correlation analysis.

### Untargeted metabolomics and analysis

One hundred microliters of serum samples were combined with 400 µL of an 80% methanol aqueous solution and vortexed. The samples were subsequently placed in an ice bath for 5 min before centrifugation at 15,000 *g* for 20 min at 4°C. The supernatant was transferred to a new tube and subsequently injected into the LC-MS/MS system for analysis. To evaluate analytical variability, equal volume aliquots from each experimental sample were pooled as quality control samples.

The serum metabolic profile was analyzed using a Vanquish UHPLC system (Thermo Fisher, Germany) coupled with an Orbitrap Q Exactive HF mass spectrometer (Thermo Fisher, Germany). The parameters for the liquid chromatography were as follows: a Hypersil Gold column (C18) was used; the mobile phase in positive and negative ion modes consisted of 0.1% formic acid in water (A) and 0.1% formic acid in methanol (B). Metabolites were eluted using the following gradient: 2% B for 1.5 min; 2%–85% B at 3 min; 85%–100% B at 10 min; 100%–2% B at 10.1 min; and 2% B at 12 min. The raw data files generated by LC-MS/MS were processed using Compound Discoverer 3.0 (CD3.3, Thermo Fisher) for peak alignment, peak selection, and quantification of each metabolite.

Qualitative and quantitative analyses of the metabolites in the samples were conducted based on the local metabolic database. Annotation of the identified metabolites was performed using the KEGG database (https://www.genome.jp/kegg/pathway.html), HMDB database (https://hmdb.ca/metabolites), and LIPID MAPS database (http://www.lipidmaps.org/).

Then metabolic data were analyzed using an OPLS-DA with the R package 'ropls' (version 1.26.4) to compare the metabolic profiles of serum samples from the NN, HFD_Abx, and HFD groups (95). To identify shared differential metabolites between the NN and HFD_Abx compared to HFD group, the two-tailed Wilcoxon rank-sum test and Maaslin2 analysis were performed, with the latter utilizing the R package 'Maaslin2' (version 1.12.0) (96). Common metabolites identified by both methods were further analyzed using Venn analysis. KEGG enrichment pathway analysis was conducted utilizing MetaboAnalyst 4.0 (http://www.metaboanalyst.ca/). To explore the relationships between differential metabolites and pubertal development, the Mantel test with the R package 'ggcor' (version 0.1.4.1) and CCpnA with the R package 'mixOmics' (version 6.22.0) were conducted (97).

### Multiomics analyses

To identify candidate biomarkers among differentially abundant microbes and metabolites, we employed DIABLO with the R package 'mixOmics' (version 6.22.0) (97). This method integrated data from both the gut microbiome and metabolomes, allowing for the identification of the main contributors from each omics in two comparison groups (HFD/NN, HFD/HFD_Abx). Prior to the DIABLO integration analysis, metabolite data were log-transformed, while microbial abundance was normalized through centered log-ratio transformation using the R package 'microbiome' (version 1.20.0) (98). The latent component number was set to 2, and the optimal number of key features in each data set was determined by ensuring minimal classification error. The relationships between candidate biomarkers and pubertal development indices were elucidated through Spearman’s rank correlation analysis.

### Statistical analysis

This study utilized R 4.2.2 software for statistical analysis. Continuous data were compared between groups using the Wilcoxon rank-sum test and presented as mean ± standard deviation. Categorical data were analyzed using the Chi-squared test or Fisher’s exact test. The *P*-values for multiple comparisons were corrected using the Benjamini-Hochberg method.

## Data Availability

The original data have been deposited at the National Omics Data Encyclopedia (NODE; https://www.biosino.org/node/index) with the accession number OEP00005841 (https://www.biosino.org/node/experiment/detail/OEX00029637).

## SUPPLEMENTAL MATERIAL

Figure S1 spectrum.03264-24-s0001.pdf The role of gut microbiota in HFD-induced precocious puberty.

Figure S2 spectrum.03264-24-s0002.pdf Abx treatment modulates the composition of gut microbiota in the HFD group.

Figure S3 spectrum.03264-24-s0003.pdf Changes in serum metabolites accompanied by alterations in gut microbiota.

Figure S4 spectrum.03264-24-s0004.pdf Correlations between gut microbiome and metabolomics profile.

Supplemental legends spectrum.03264-24-s0005.docx Legends for Fig. S1 to S4 and Table S1 to S8.

Tables S1 to S8 spectrum.03264-24-s0006.xlsx Differences in the abundance of pubertal development indicators, microbial taxa, functional pathways, and metabolites between the HFD vs. NN groups and the HFD vs. HFD_Abx groups were analyzed, along with Spearman correlation analysis.

ASM does not own the copyrights to Supplemental Material that may be linked to, or accessed through, an article. The authors have granted ASM a non-exclusive, world-wide license to publish the Supplemental Material files. Please contact the corresponding author directly for reuse.
