# Supplementary figures and images for: Gut microbiota alterations modulate high-fat diet-induced precocious puberty
Source: Microbiol Spectr. 2025 Aug 12;13(9):e03264-24. doi: 10.1128/spectrum.03264-24 (PMC12403903; doi:10.1128/spectrum.03264-24)

A

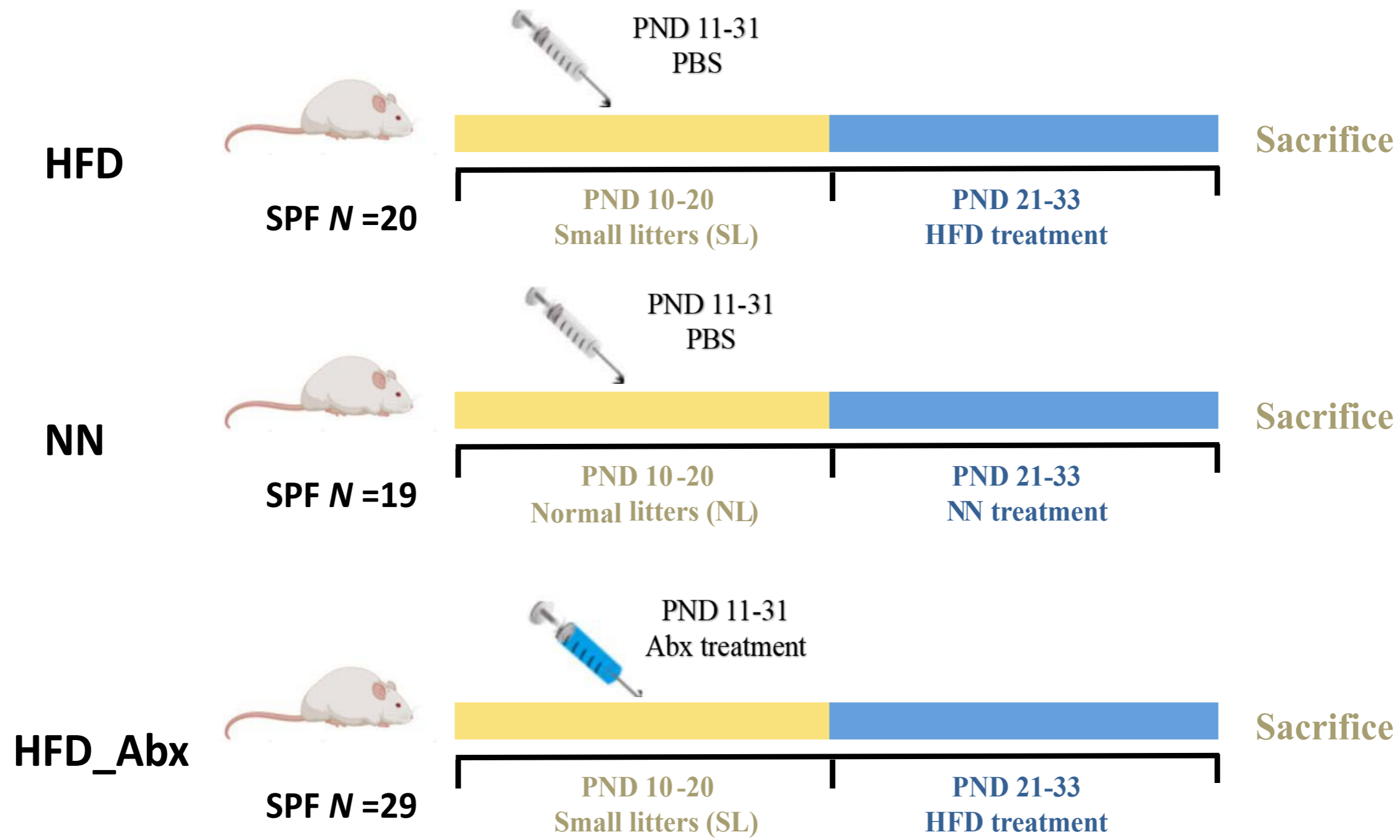

B

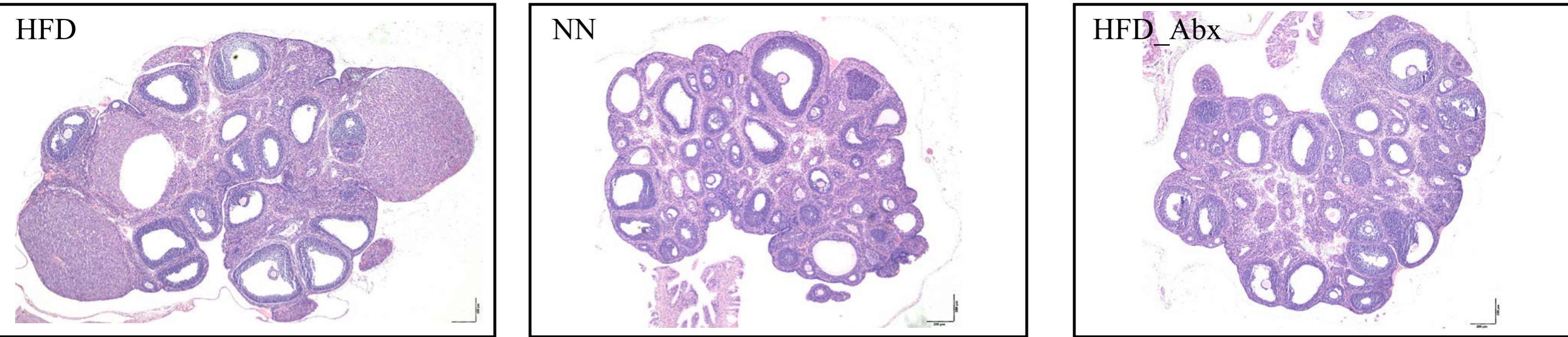

Supplement: Figure S1 — The role of gut microbiota in HFD-induced precocious puberty. [file spectrum.03264-24-s0001.pdf]

A

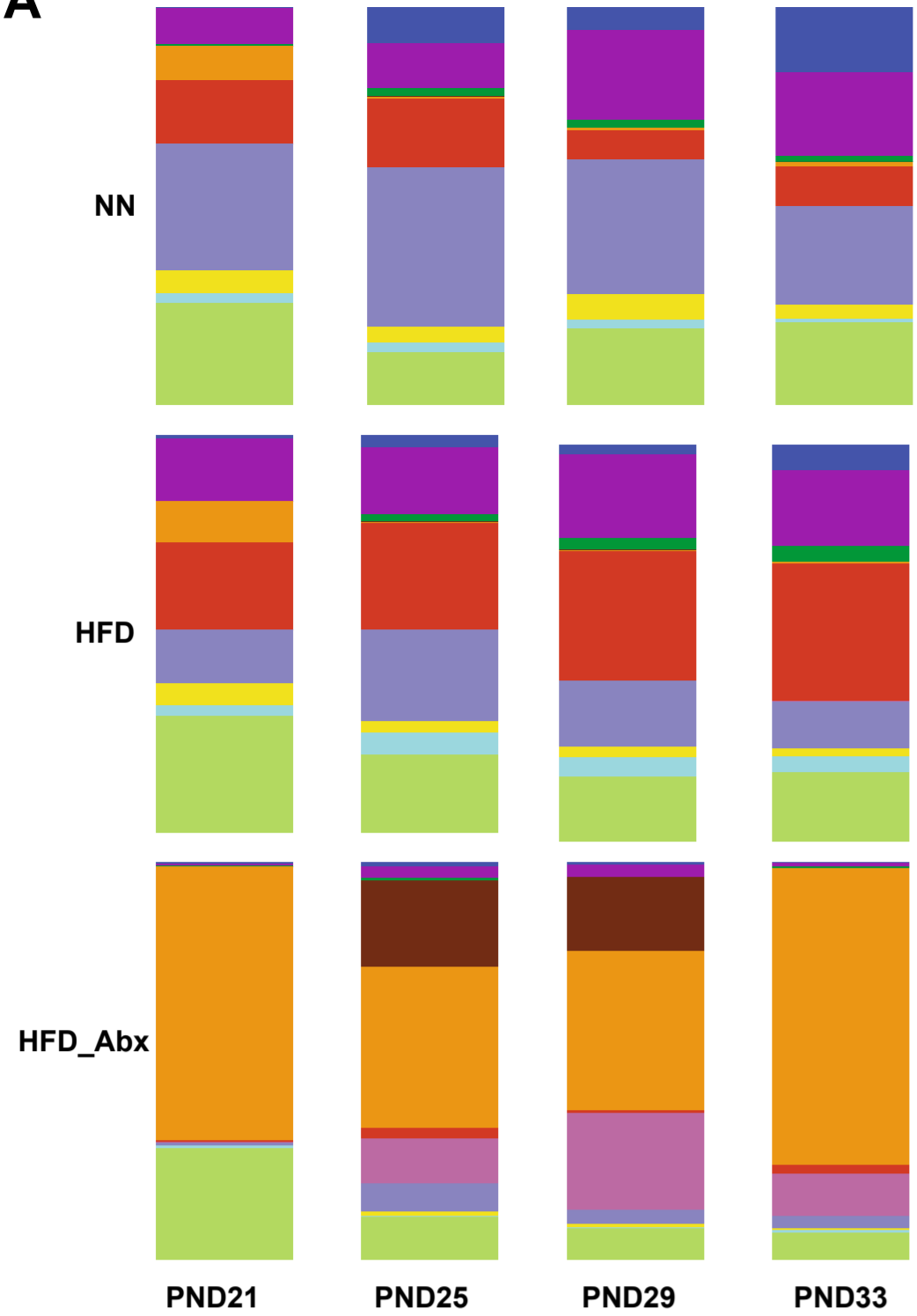

B

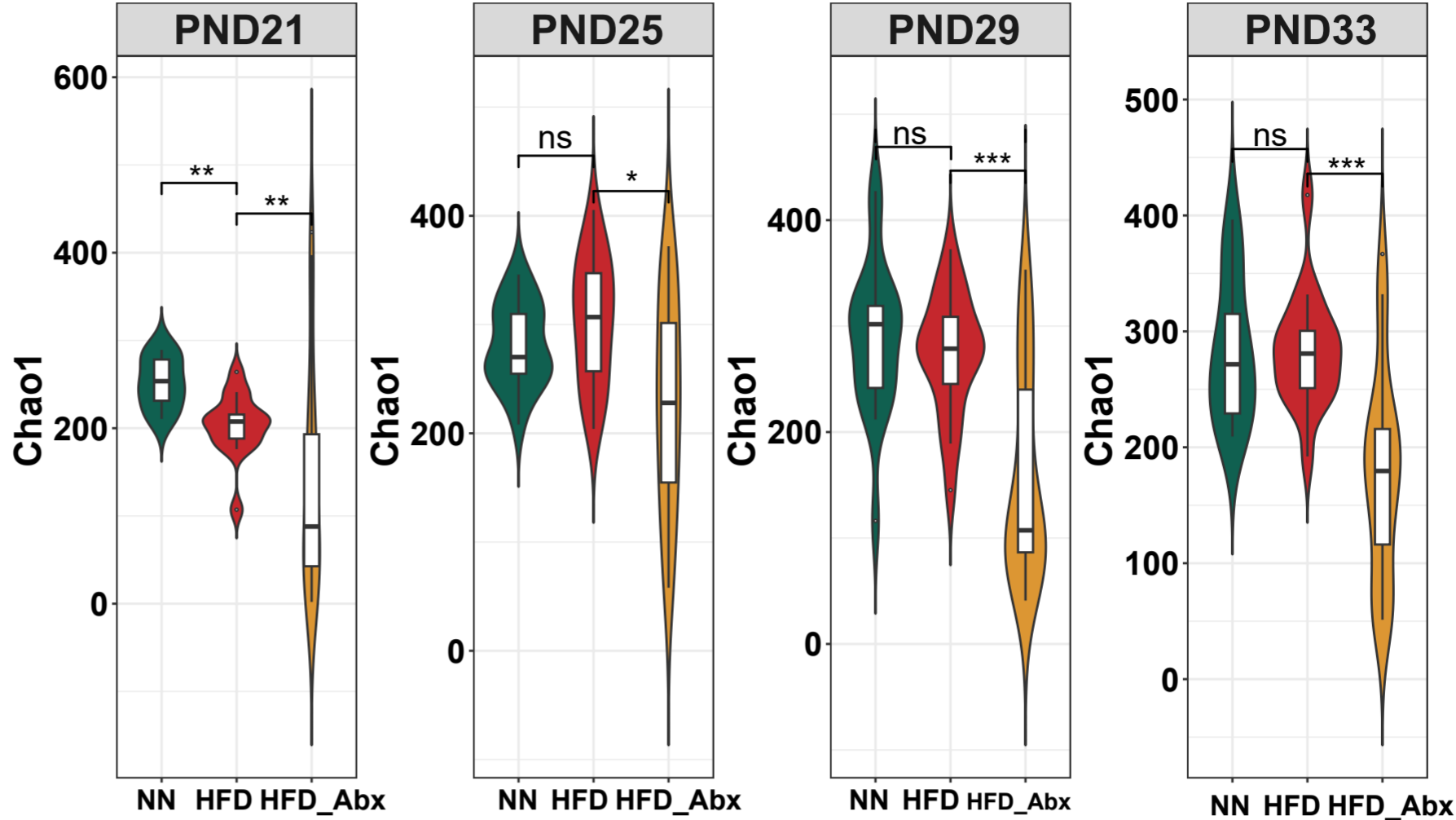

C

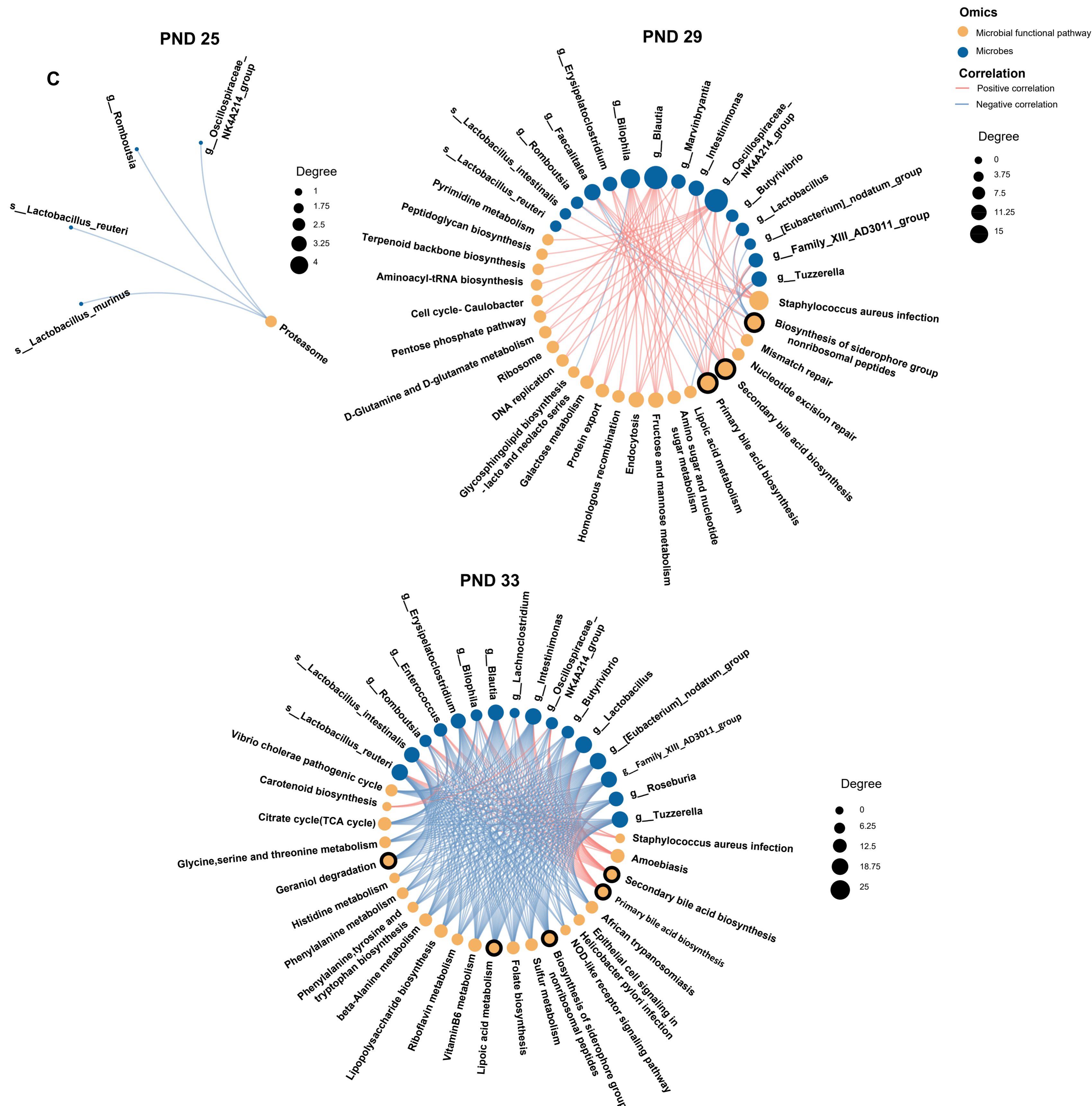

Supplement: Figure S2 — Abx treatment modulates the composition of gut microbiota in the HFD group. [file spectrum.03264-24-s0002.pdf]

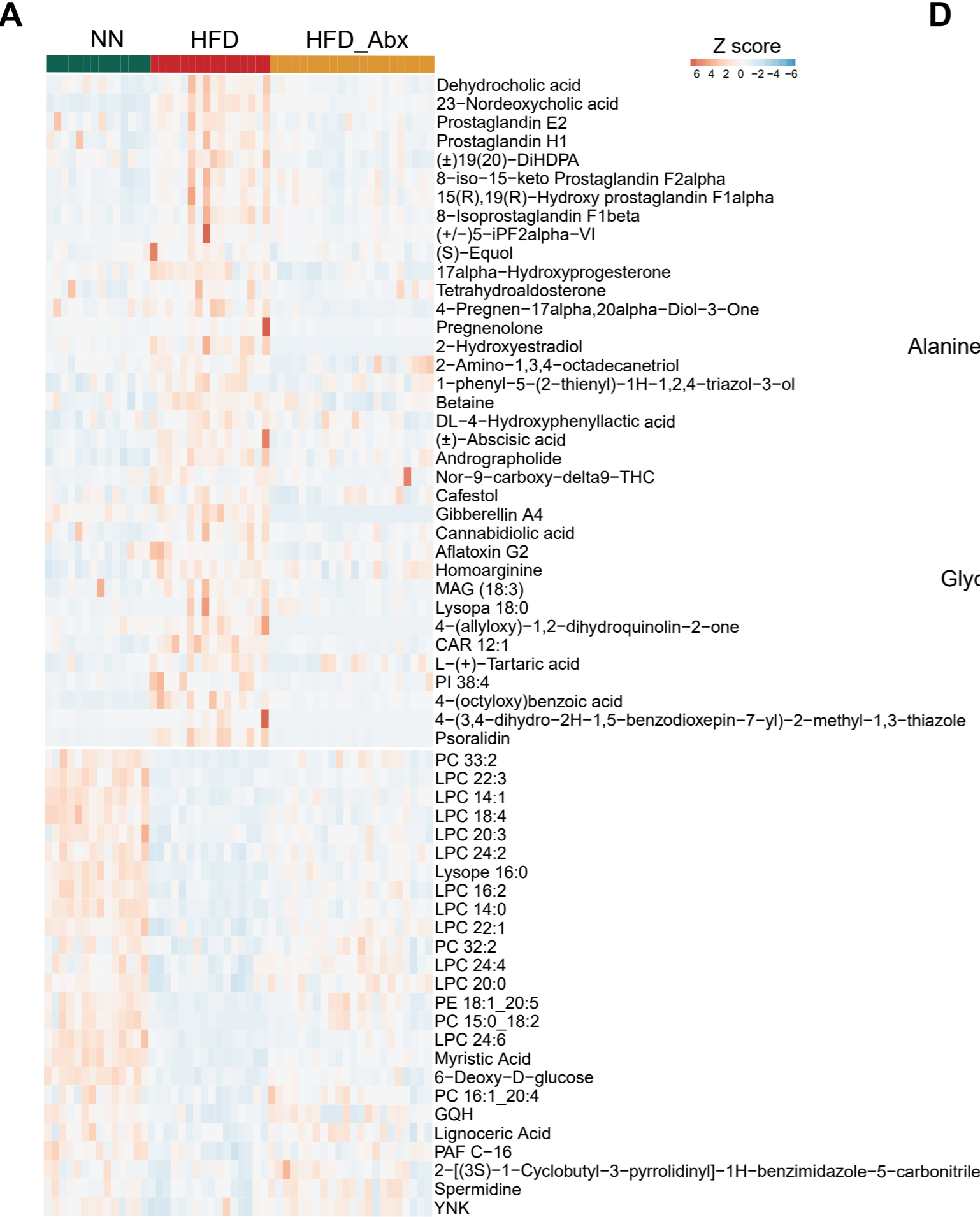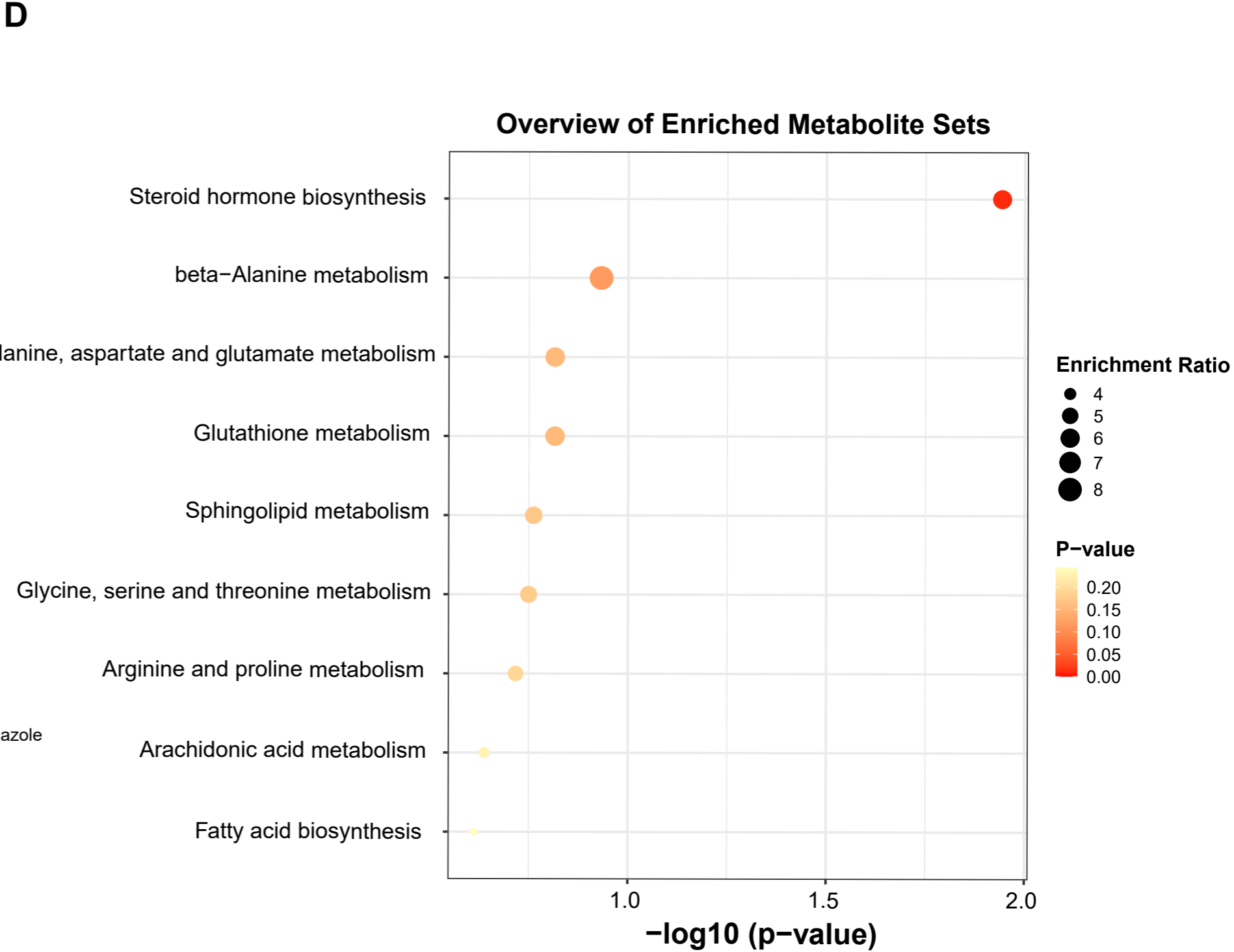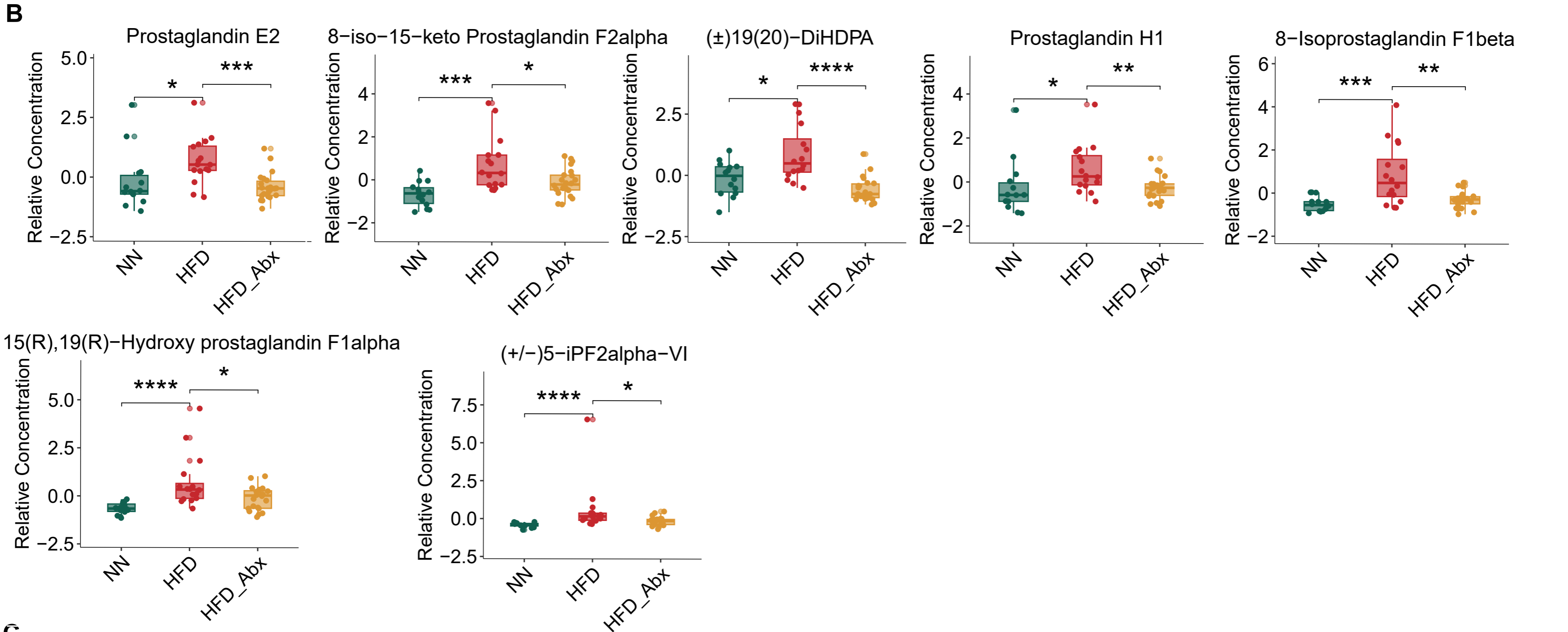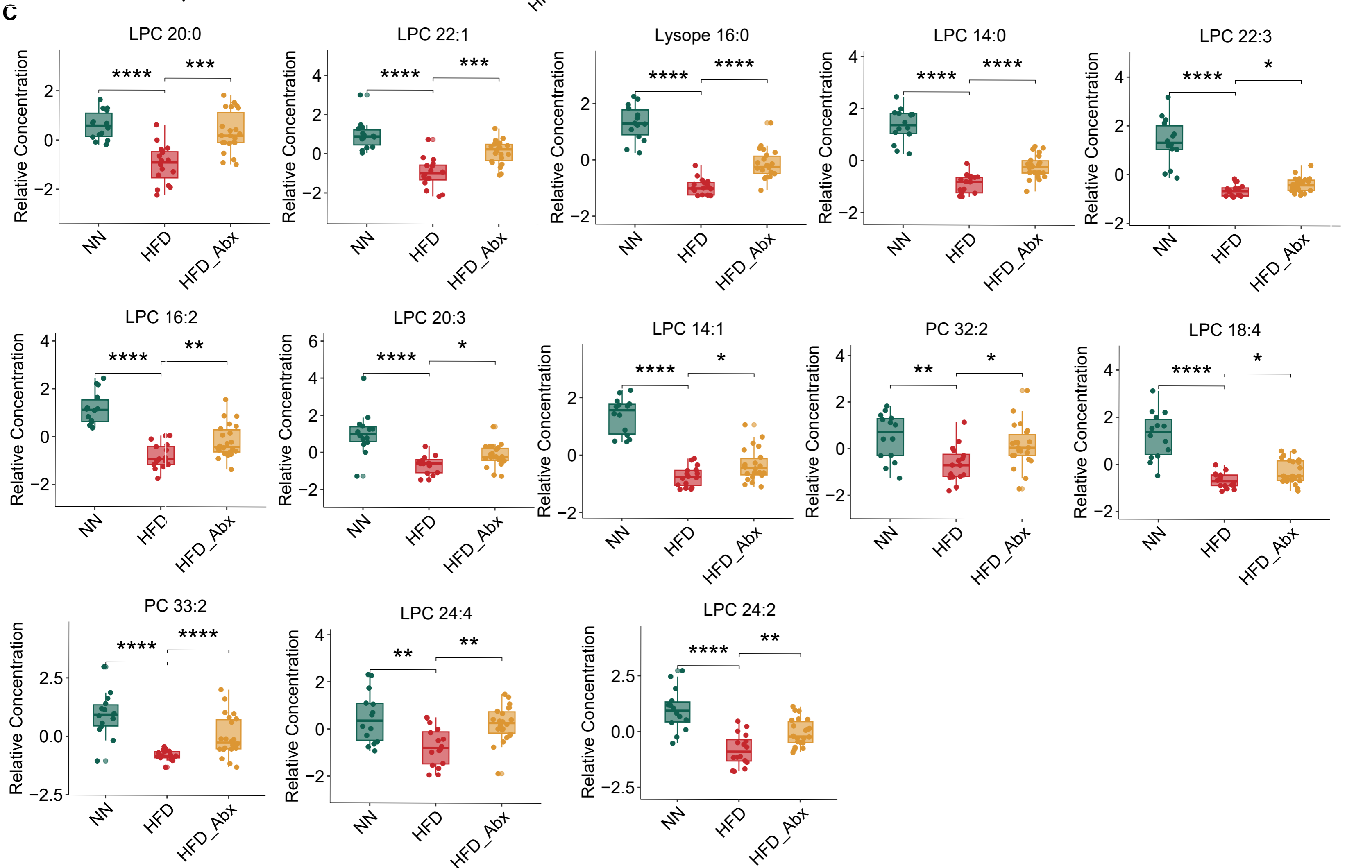

Supplement: Figure S3 — Changes in serum metabolites accompanied by alterations in gut microbiota. [file spectrum.03264-24-s0003.pdf]

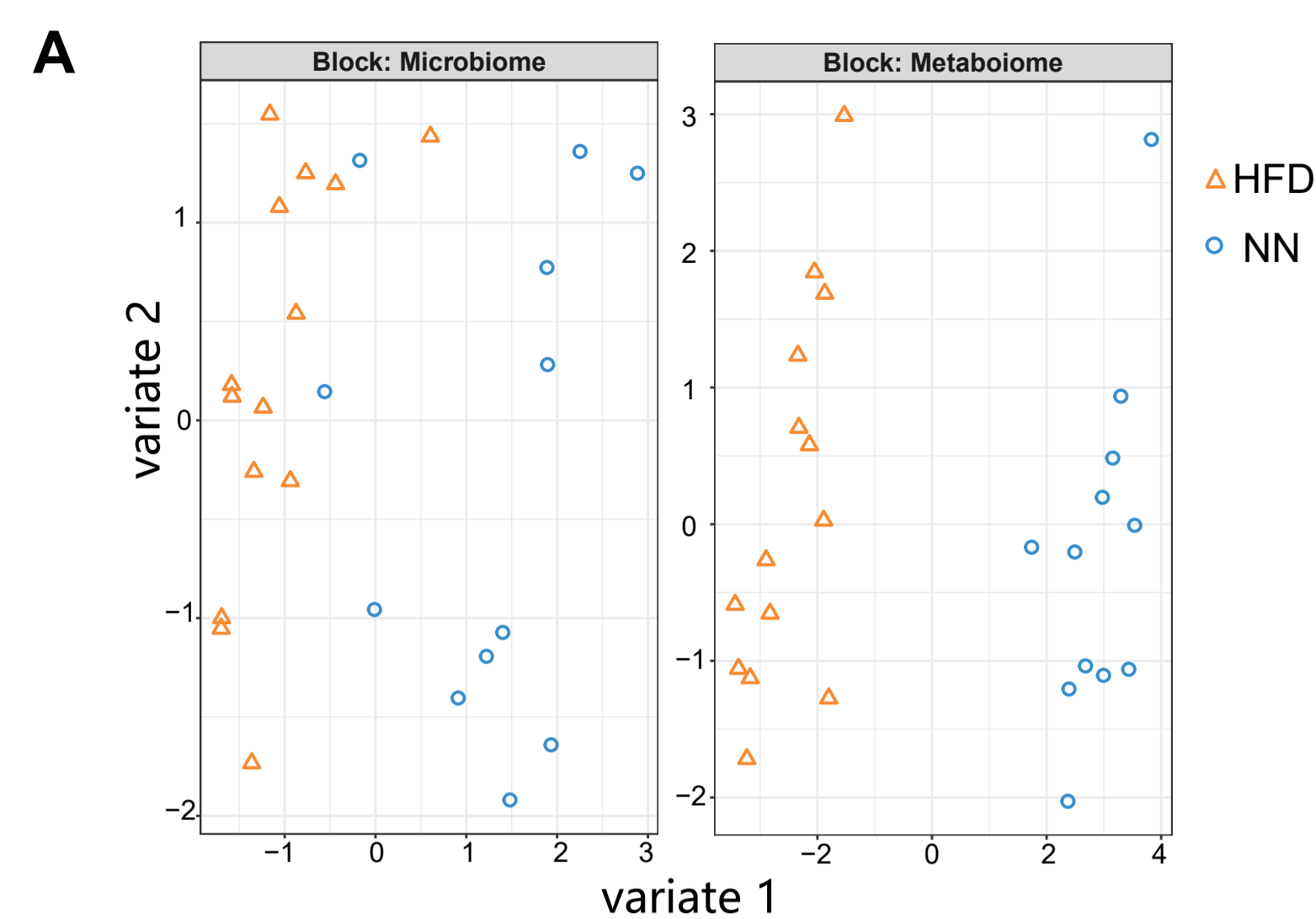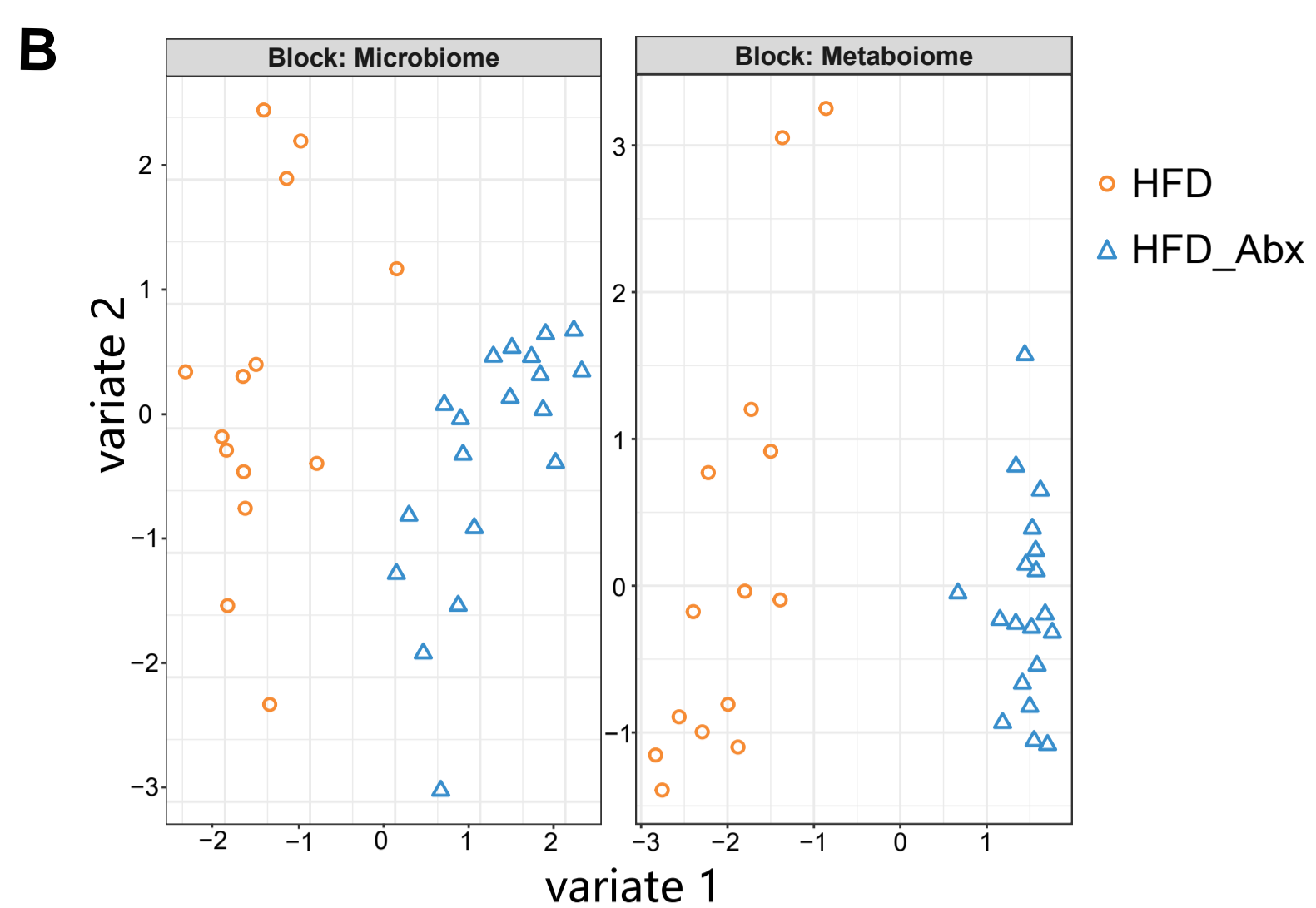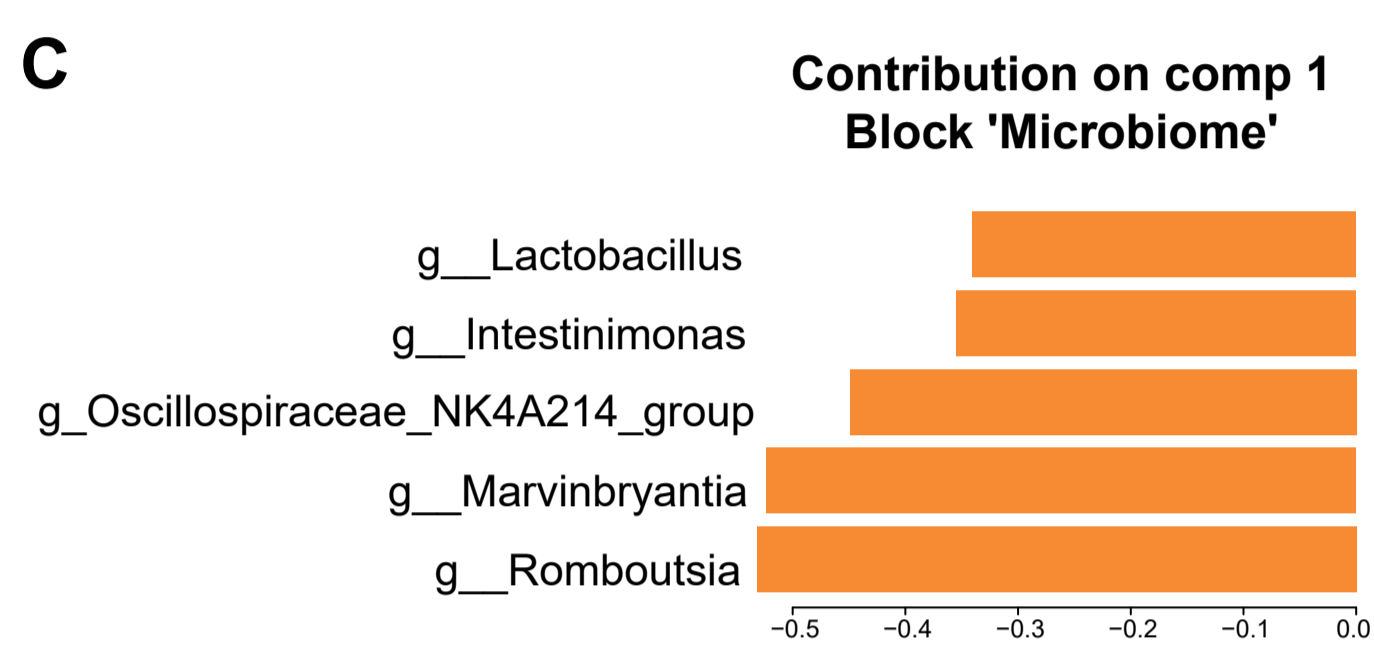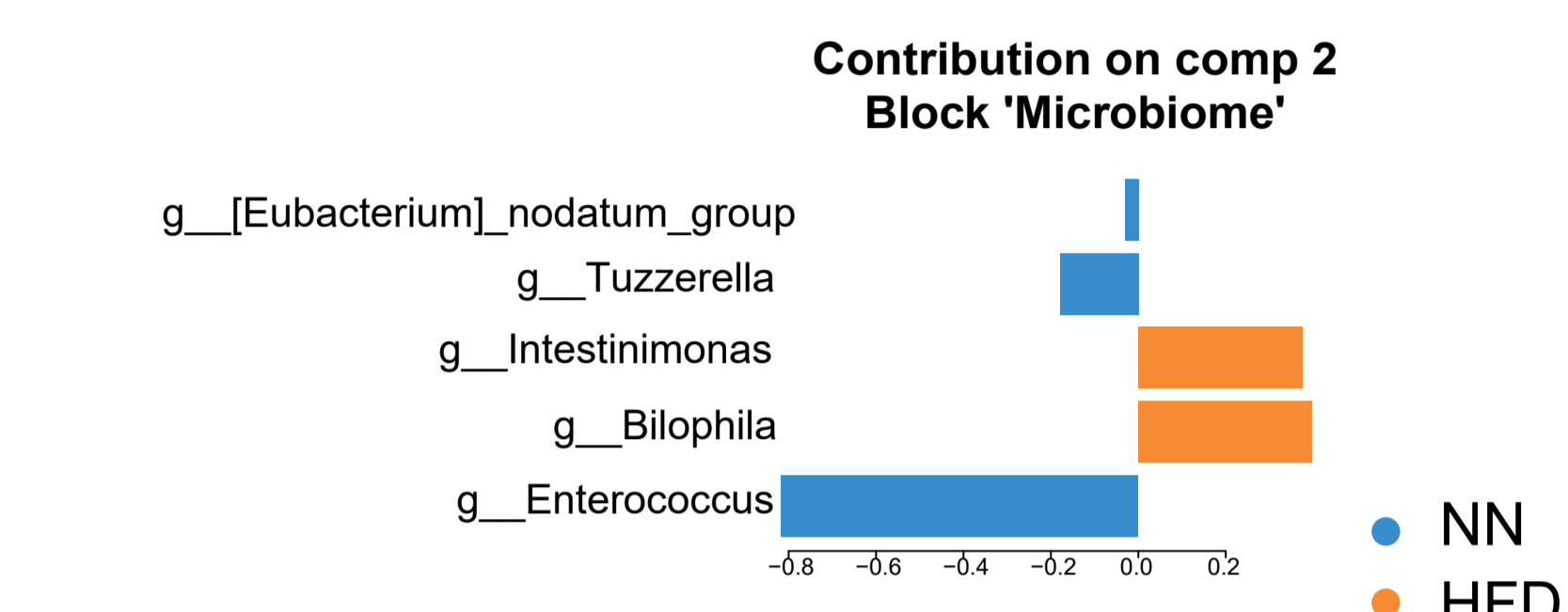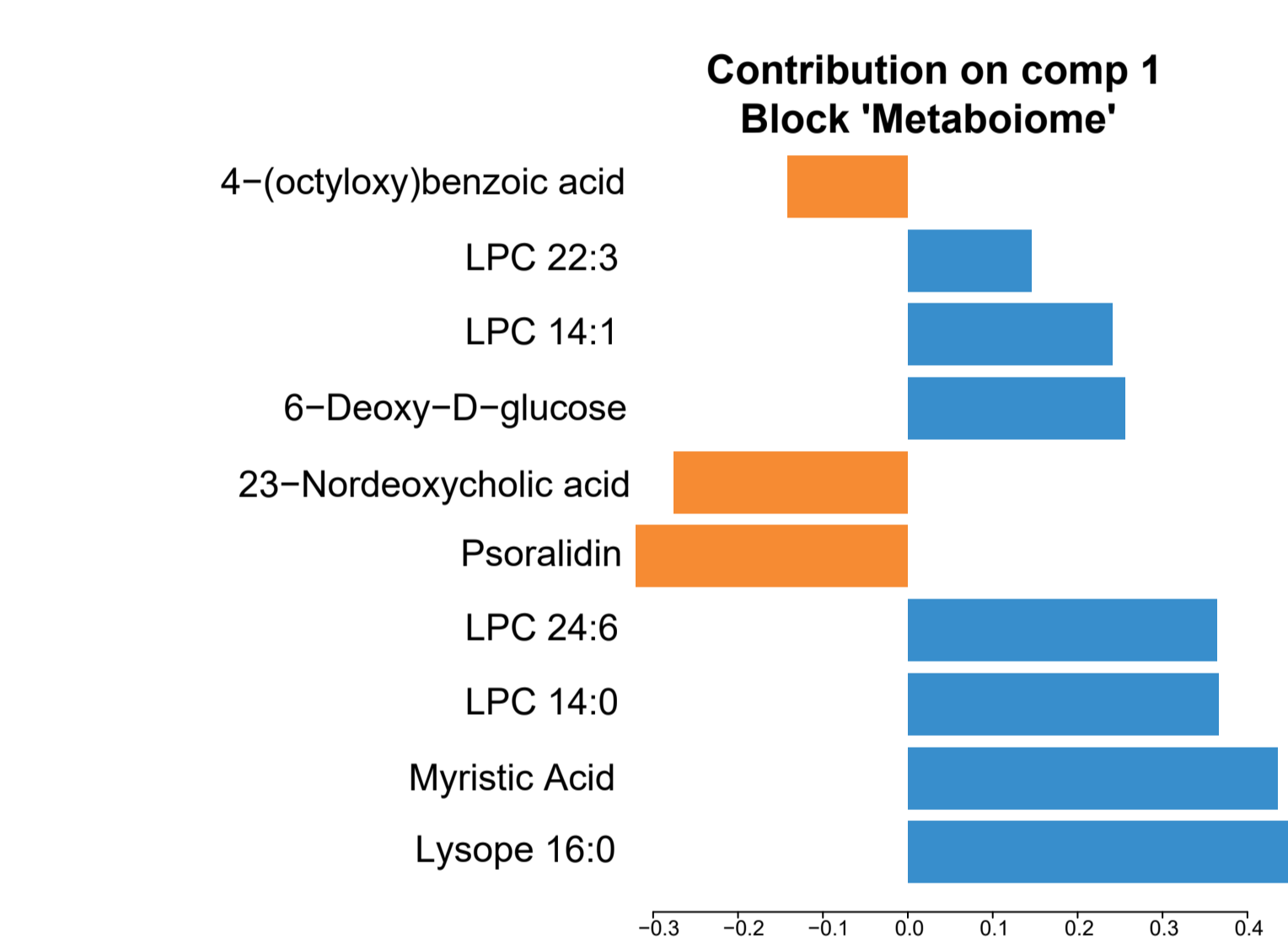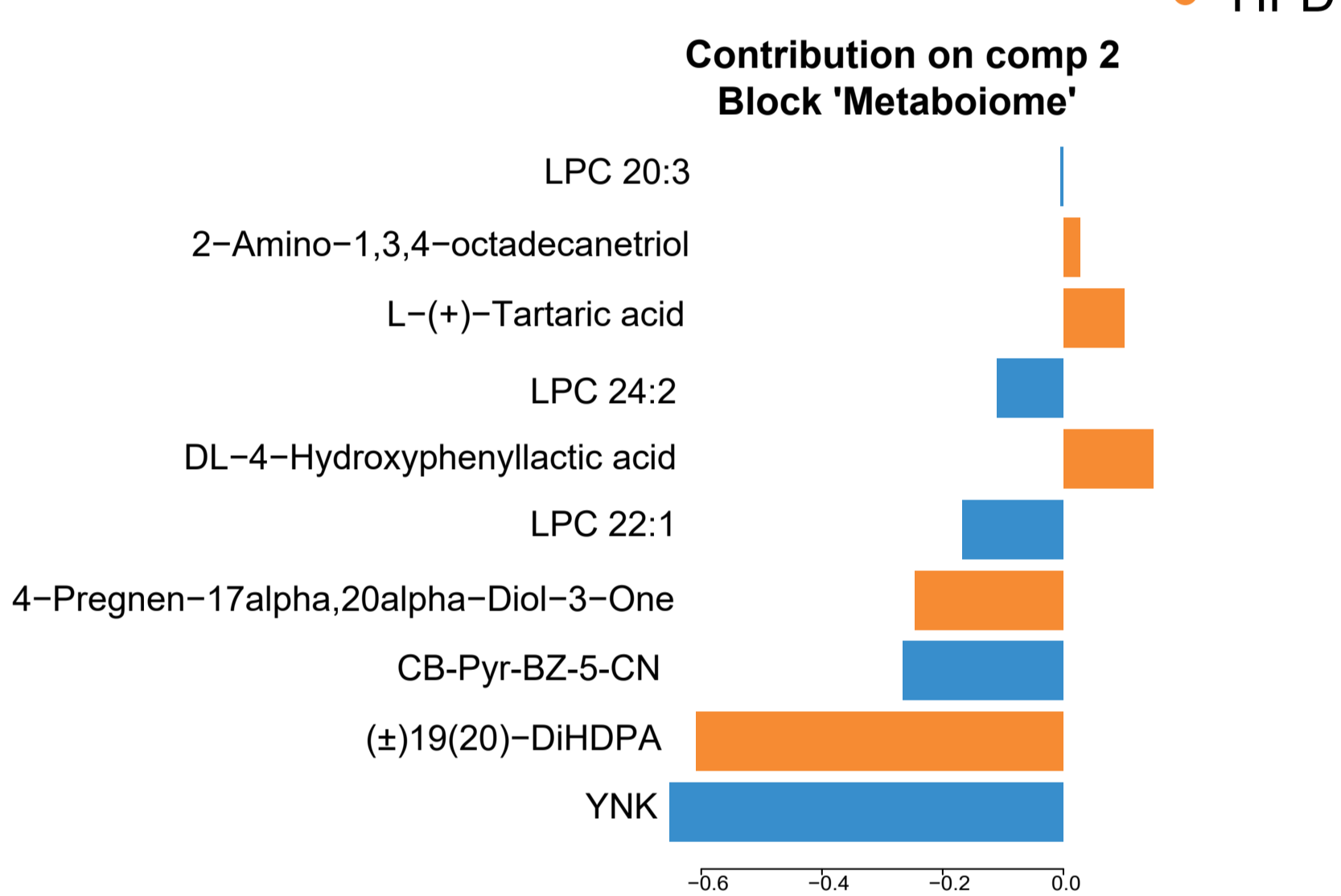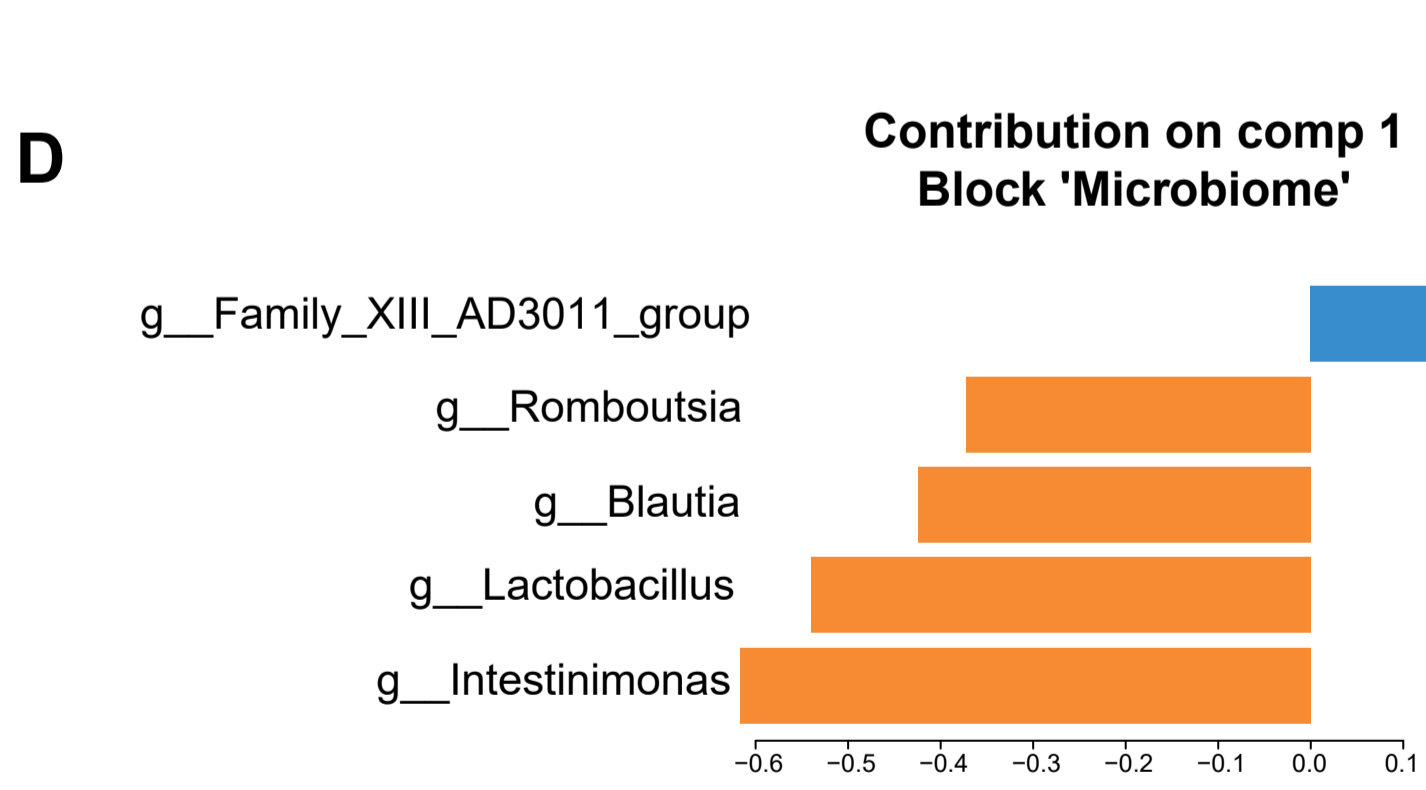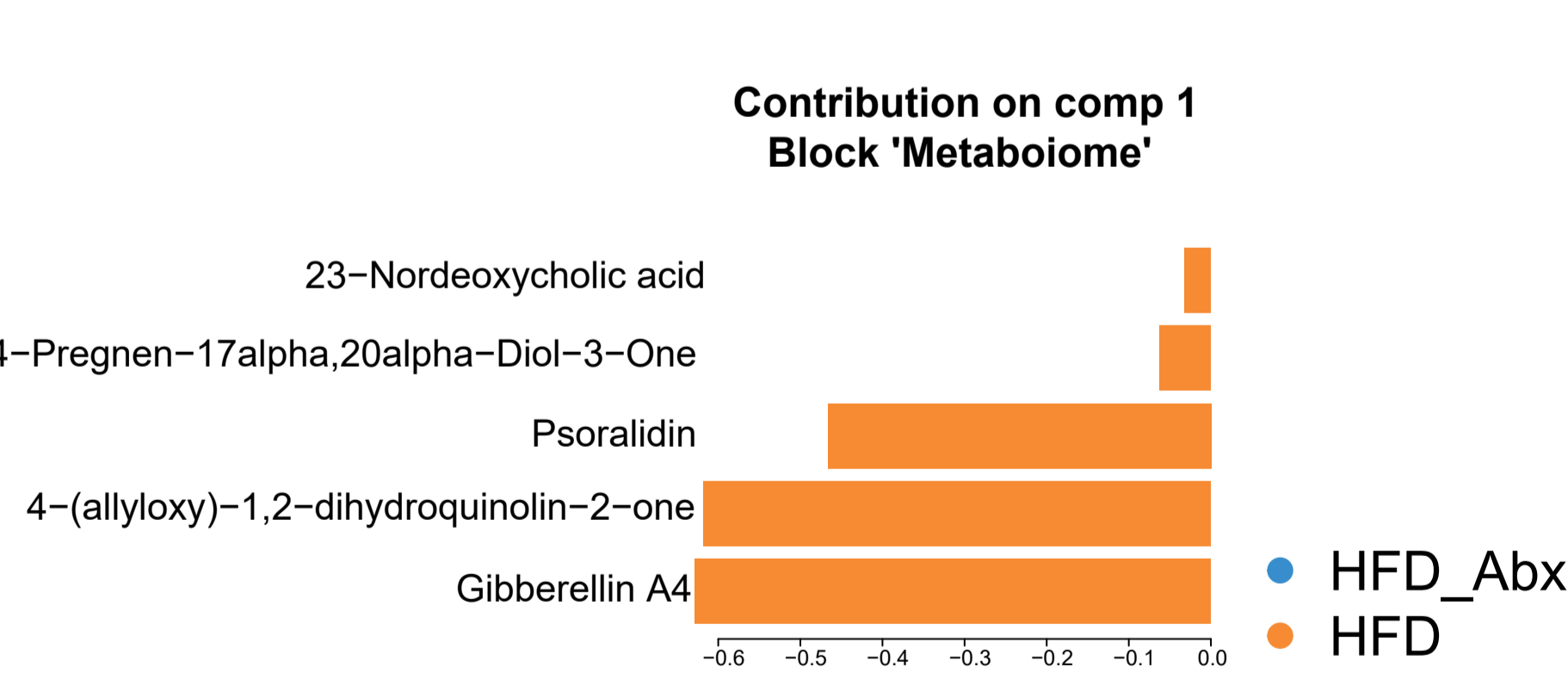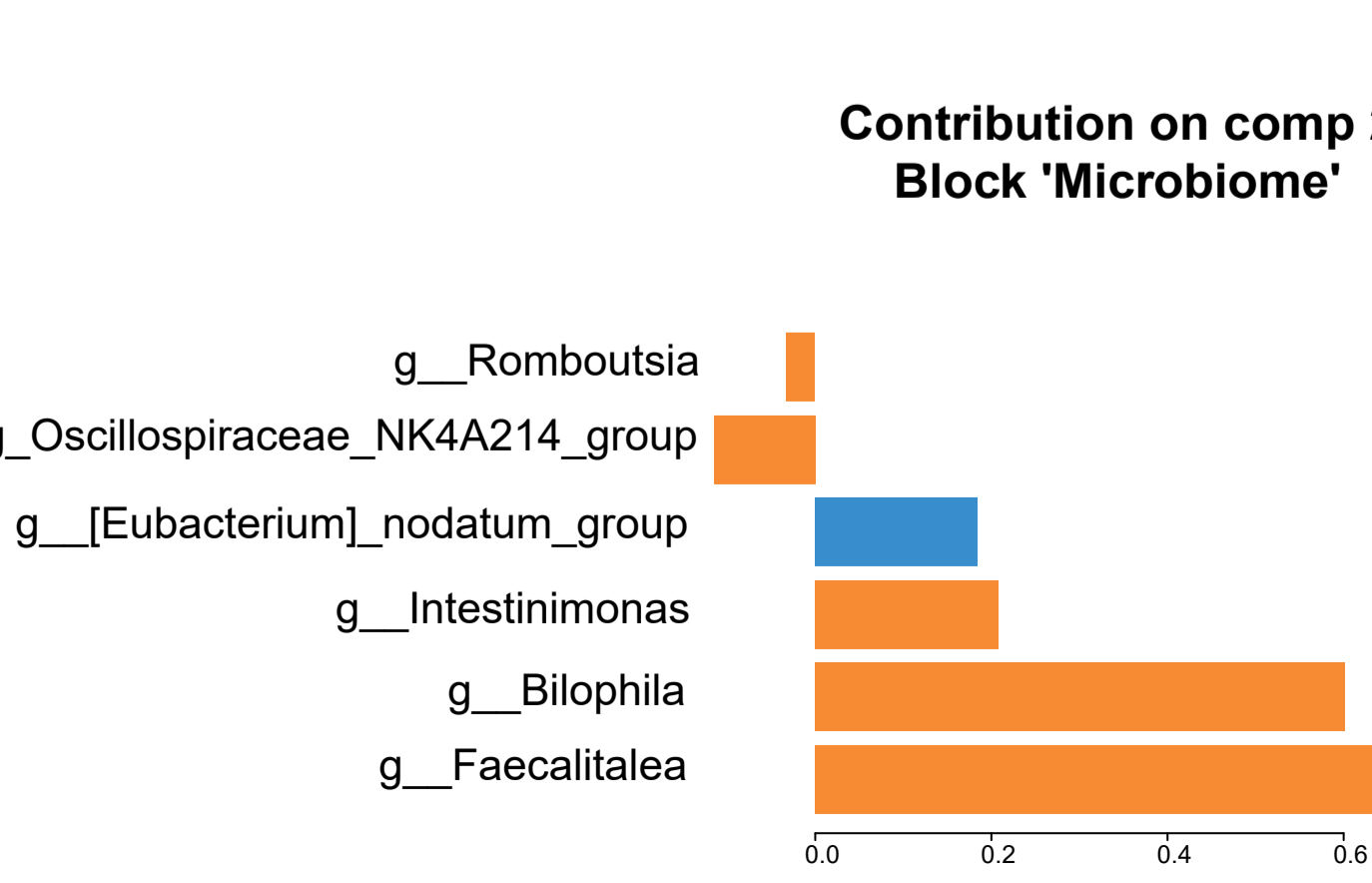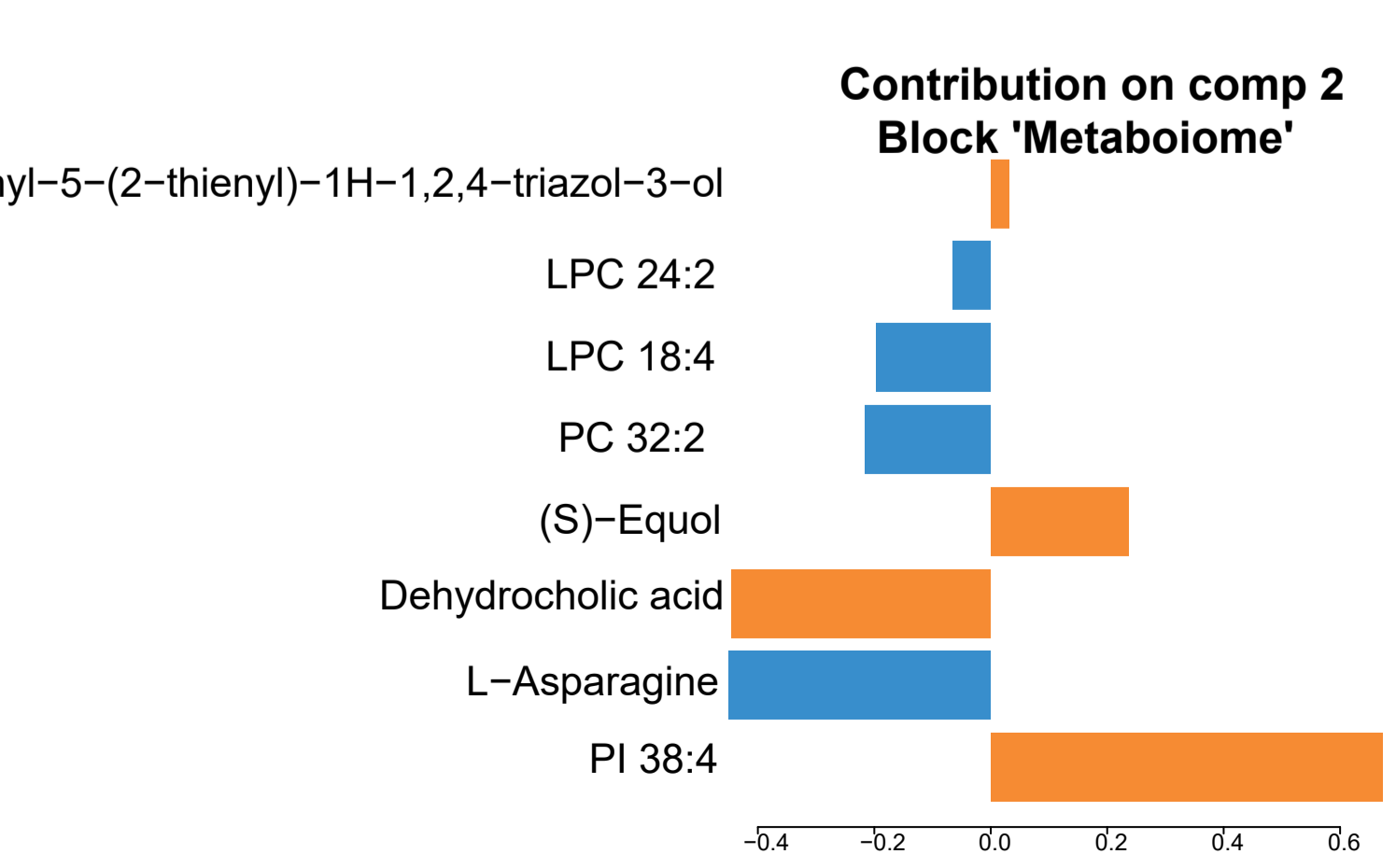

Supplement: Figure S4 — Correlations between gut microbiome and metabolomics profile. [file spectrum.03264-24-s0004.pdf]
