# Supplementary material for: Gut microbiota alterations modulate high-fat diet-induced precocious puberty
Source: Microbiol Spectr. 2025 Aug 12;13(9):e03264-24. doi: 10.1128/spectrum.03264-24 (PMC12403903; doi:10.1128/spectrum.03264-24)
Supplement: Supplemental legends — Legends for Fig. S1 to S4 and Table S1 to S8. [file spectrum.03264-24-s0005.docx]

**Supplementary Material**

**Separate supplementary figures and tables - Legends**

Figure S1. The role of gut microbiota in HFD-induced precocious puberty. A. The experimental protocol for assessing the role of gut microbiota in precocious puberty induced by HFD in female rats. Female rats fed HFD were administered either an antibiotic cocktail (HFD_Abx) or a PBS (HFD) for three weeks. Rats that were treated with a standard diet combined with PBS served as the control group (NN). B. Change in ovarian maturity.

Figure S2. Abx treatment modulates the composition of gut microbiota in the HFD group. A. The microbial composition at the genus levels in PND 21, PND 25, PND 29, and PND 33. B. α-diversity (Chao1 index) of the gut microbiome in the HFD, HFD_Abx and NN groups at four timepoints. C. Spearman correlations between 19 identified bacteria and different microbial functional pathways in three groups at three timepoints.

Figure S3. Changes in serum metabolites accompanied by alterations in gut microbiota. A. The abundances of the shared 62 differential metabolites. Comparison of PGs and their derivatives (B), and GPs (C) between the three groups. D. The enrichment analysis of KEGG metabolic pathways according to differential metabolites. The two-tailed Wilcoxon rank-sum test (A-B) and Maaslin2 (A) analyses were used for statistical analysis with Benjamini-Hochberg adjustment. **adj.p* < 0.05; ***adj.p* < 0.01; ****adj.p* < 0.001; *****adj.p* < 0.0001.

Figure S4. Correlations between gut microbiome and metabolomics profile. Scatter plots showing clear discrimination of samples according to the features of gut microbiome (left) and metabolomics (right) selected by the integrated multi-omics DIABLO, for HFD vs. NN (A) and HFD vs. HFD_Abx (B). Loading plots of the bacteria and metabolites with maximum contributions on component 1 and component 2, which was calculated by DIABLO between HFD and NN groups (C) as well as HFD and HFD_Abx groups (D). CB-Pyr-BZ-5-CN, 2-[(3S)-1-cyclobutyl-3-pyrrolidinyl]-1H-benzimidazole-5-carbonitrile.

Table S1 Indicators of pubertal development differing between HFD vs. NN and HFD vs. HFD_Abx (Wilcoxon).

Table S2 Shared taxa differing between HFD vs. NN and HFD vs. HFD_Abx (ZIBR).

Table S3 Taxa differing between HFD vs. NN and HFD vs. HFD_Abx (Wilcoxon).

Table S4 Microbial functional pathways differing between the two comparison groups (Wilcoxon).

Table S5 Shared differentially abundant metabolites between the two comparison groups (Wilcoxon).

Table S6 Shared differentially abundant metabolites between the two comparison groups (MaAsLin2).

Table S7 Spearman correlation analysis.

Table S8 The primer sequence of different genes in RT-PCR.
